# A Detailed View on the (Re)isomerization Dynamics in Microbial Rhodopsins Using Complementary Near‐UV and IR Readouts

**DOI:** 10.1002/anie.202416742

**Published:** 2024-11-26

**Authors:** Marvin Asido, Gerrit H. U. Lamm, Jonas Lienert, Mariafrancesca La Greca, Jagdeep Kaur, Anne Mayer, Clemens Glaubitz, Joachim Heberle, Ramona Schlesinger, Kirill Kovalev, Josef Wachtveitl

**Affiliations:** ^1^ Institute of Physical and Theoretical Chemistry Goethe University Frankfurt Max-von-Laue Straße 7 60438 Frankfurt (Main) Germany; ^2^ Present Adress: Department of Chemistry Massachusetts Institute of Technology 77 Massachusetts Ave, 2–014 Cambridge Massachusetts 02139 USA; ^3^ Department of Physics Genetic Biophysics Freie Universität Berlin Arnimallee 14 14195 Berlin Germany; ^4^ Institute for Biophysical Chemistry and Center for Biomolecular Magnetic Resonance (BMRZ) Goethe University Frankfurt Max-von-Laue Straße 9 60438 Frankfurt (Main) Germany; ^5^ Department of Physics Experimental Molecular Biophysics Freie Universität Berlin Arnimallee 14 14195 Berlin Germany; ^6^ European Molecular Biology Laboratory Hamburg, EMBL Hamburg c/o DESY, Notkestraße 85 22607 Hamburg Germany

**Keywords:** energy conversion, IR spectroscopy, photoisomerization, microbial rhodopsins, UV/Vis spectroscopy

## Abstract

Isomerization is a key process in many (bio)chemical systems. In microbial rhodopsins, the photoinduced isomerization of the all‐*trans* retinal to the 13‐*cis* isomer initiates a cascade of structural changes of the protein. The interplay between these changes and the thermal relaxation of the isomerized retinal is one of the crucial determinants for rhodopsin functionality. It is therefore important to probe this dynamic interplay with chromophore specific markers that combine gapless temporal observation with spectral sensitivity.

Here we utilize the near‐UV and mid‐IR fingerprint region in the framework of a systematic (time‐resolved) spectroscopic study on H^+^‐ (*Hs*BR, (G)PR), Na^+^‐ (KR2, *Er*NaR) and Cl^−^‐(*Nm*HR) pumps. We demonstrate that the near‐UV region is an excellent probe for retinal configuration and—being sensitive to the electrostatic environment of retinal—even transient ion binding, which allows us to pinpoint protein specific mechanistic nuances and chromophore‐charge interactions. The combination of the near‐UV and mid‐IR fingerprint region hence provides a spectroscopic analysis tool that allows a detailed, precise and temporally fully resolved description of retinal configurations during all stages of the photocycle.

## Graphical abstract

The near‐UV readout (SBS) in microbial rhodopsins can be used to probe the retinal configuration as well as electrostatic changes associated with the charge translocation during the ion pumping process in real time.

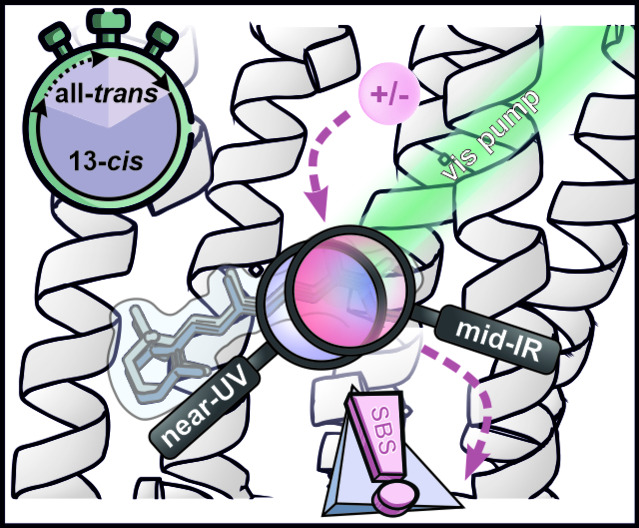

## Introduction

**Figure 1:**
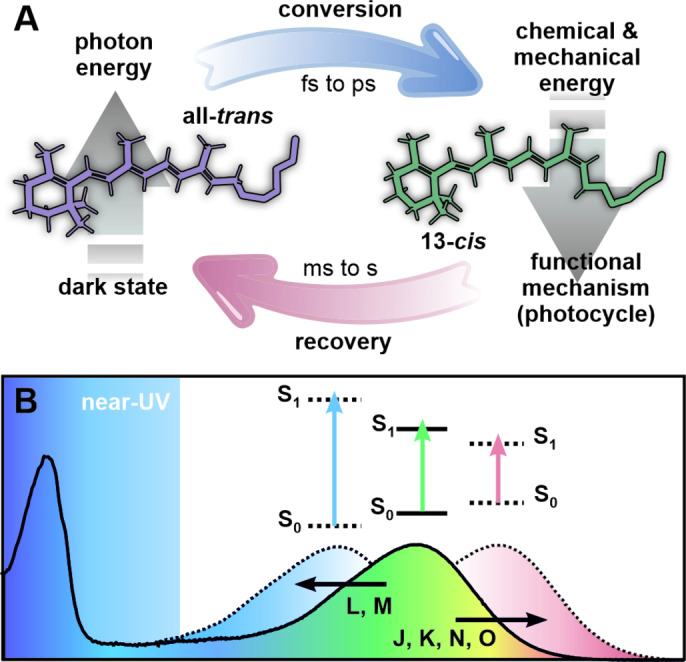
(A) Simplified scheme of the retinal‐associated energy conversion, starting with the absorption of a photon. The initial energy is successively used for structural rearrangements of both the chromophore and the protein or dissipated as heat during the photocycle. (B) Spectral shifts due to electrostatic changes around the retinal throughout the photocycle. The main absorption band stems from the strong CT character of the S_0_–S_1_ transition of retinal. The near‐UV region spans the spectral gap between the blue shifted absorption bands of retinal and the strong absorption of aromatic amino acid residues in the protein backbone.

The direct conversion of light into chemical and/or mechanical energy has been one of the key processes in the development and evolution of life on earth and is now equally important for the rising demand in modern society to reconcile economic growth and global sustainability. Photoisomerization is such a process which converts a photon into molecular motion and hence drives the (reversible) configurational change from isomer A to isomer B of the molecule. A broad variety of such so‐called photoswitches and their derivatives have been well studied and implemented in systems with varying degrees of complexity. Yet, many biological systems found in nature have adapted and evolutionarily optimized this concept in many cases to unmatched efficiencies by combining a photoswitchable co‐factor with an apoprotein of defined function, making the unit photoresponsive. Microbial rhodopsins represent one of the largest classes of such light‐sensitive proteins and are therefore responsible for a significant amount of light‐to‐chemical‐energy conversion in a wide variety of ecosystems.[^1^, ^2^, ^3^] The specific functionality of a microbial rhodopsin can vary in terms of the mode (ion channel, proton pump, cation pump or anion pump) and the direction (inward vs. outward) of ion conductance across a biological membrane.[^1^, ^2^, ^3^] Nonetheless, all microbial rhodopsins share common structural features as a whole and very nuanced structural motifs amongst their respective subclasses.[^1^, ^2^, ^3^] All microbial rhodopsins consist of the opsin unit (with seven transmembrane *α*‐helices) and the light‐sensitive retinal chromophore which is attached to the apoprotein by a Schiff base linkage.[^1^, ^2^, ^3^] This retinal Schiff base (RSB) is crucial for the accessibility and the direction of the translocated ions.^[4]^ The required energy for the protein functionality is harnessed by the chromophore which—upon absorption of a photon—isomerizes from the all‐*trans* to the 13‐*cis* configuration.[^5^, ^6^, ^7^, ^8^] The photoisomerization induces a cascade of structural changes in the protein which are usually intertwined with further changes of the retinal (Figure 1). This interplay between the dynamic adjustments of the protein and the thermal recovery of retinal itself is considered a key factor for the diverse functionalities amongst microbial rhodopsins.

According to the isomerization/switch/translocation (IST)‐model^[4]^ the configurational state of retinal determines the accessibility of RSB hydrogen and hence also the deprotonation/reprotonation steps during the photocycle which influence the vectoriality of the charge translocation. A deep understanding of the chromophore dynamics is therefore crucial to understand the overall functional mechanism of the protein. With respect to optogenetics, a major effort is put into the structural elucidation and the design of new (and structurally altered) variants with increased efficiency, responsiveness or even functionality,[^9^, ^10^] however the influence of such structural changes on the chromophore dynamics is not easy to predict and studies in this context are still underrepresented. The configurational changes of the retinal are usually transiently probed via vibrational spectroscopy (IR and Raman). The UV/vis spectral range, in contrast, delivers information about the color tuning of the chromophore (e.g. its spectrum) with its changing steric and electrostatic environment. To get a clear picture of the underlying protein‐mediated chromophore dynamics, vibrational and electronic spectroscopic methods are needed. In our previous KR2 study we discussed a near‐UV signature which corresponds to a transition to a higher electronic state (second bright state, SBS) of the 13‐*cis* retinal configuration.^[11]^ In contrast to the main transition, the SBS has no charge‐transfer(CT)‐character (from the SB nitrogen to the *β*‐ionone ring),^[12]^ and hence it shows almost no sensitivity toward electrostatic changes around the RSB (no significant spectral shifts).^[11]^ The SBS does, however, undergo a significant spectral shift correlated with the transient sodium uptake. Therefore, we extend and generalize the discussion to the prototypic light‐driven proton pumps (*Hs*BR and (G)PR—from now on simply referred to as PR)[^13^, ^14^] as well as more recently discovered light‐driven ion pumps (*Er*NaR and *Nm*HR)[^15^, ^16^] using complementary time‐resolved UV/vis and IR spectroscopy. This requires us to briefly characterize the photocycle of the respective microbial rhodopsins with a focus mainly on the chromophore (re)isomerization dynamics, its characteristic IR and/or UV/vis bands and the interconnection with the transient charge transport. Since this study includes the discussion of several proteins (some of which are already discussed abundantly in the current literature), and hence aims to establish the best possible (cross)comparability amongst them, all data were subjected to a quasi model‐free lifetime distribution analysis (LDA) to minimize model‐ and/or technique‐related bias. However, also a complete global lifetime analysis (GLA) is provided in the Supporting Information for additional reference.

## Results and Discussion

### The Near‐UV Absorption on the fs‐Timescale

**Figure 2:**
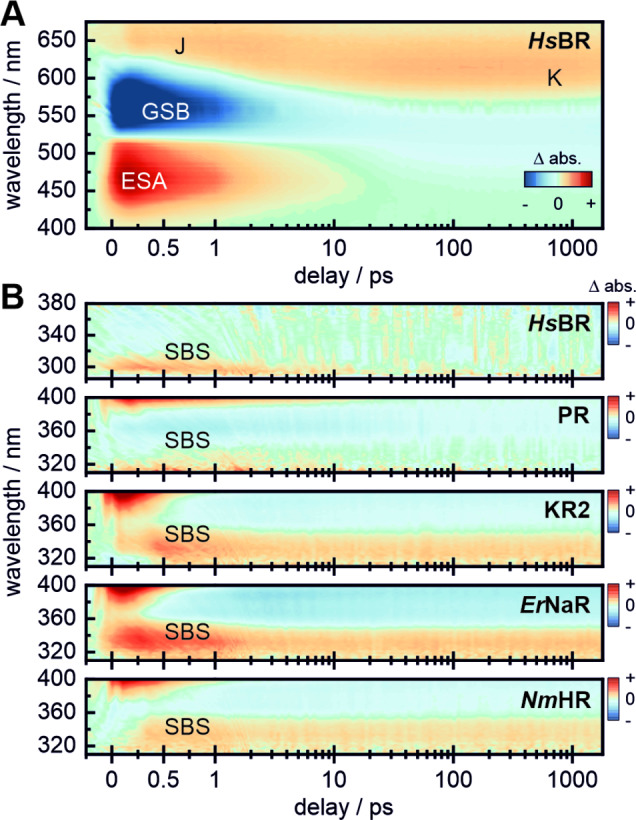
Comparative ultrafast TA measurements on five microbial rhodopsins. The *Hs*BR dataset (A) is used as a “blueprint” to introduce the typical spectroscopic signatures in the visible window. (B) Extended spectral window spanning 290–380 nm for *Hs*BR and 310–400 nm for PR, KR2, *Er*NaR and *Nm*HR, showcasing the SBS signature from the early fs‐range up to the end of the measurement window (scaled by x3 to match the amplitude of the larger signals in the visible range). The extended datasets and full lifetime analysis are provided in the Supporting Information (Figures S2–S5 and Table S2).

The absorption of a photon by the retinal chromophore leads to a multi‐phasic decay of the excited state which is usually reflected by 2–3 lifetime components in the early fs and ps regime.[^17^, ^18^, ^19^, ^20^] In essence, one can distinguish between the reactive pathway (leading to the isomerization of the retinal) and the non‐reactive pathway.[^20^, ^21^] Such reaction pathways in the excited state are determined by the topology of the potential energy surface,[^5^, ^6^] which is strongly influenced by the steric and electrostatic environment of the chromophore (i.e. hydrogen bonding at the RSB, counterion interactions, …).[^22^, ^23^, ^24^] The isomerization of retinal from all‐*trans* to 13‐*cis* (reactive pathway) occurs on the hundreds of fs timescale and is spectroscopically indicated by the formation of the red‐absorbing hot ground state (J‐intermediate) which relaxes thermally to form the stable photoproduct (K‐intermediate) on the ps timescale (Figure 2A and S3).[^5^, ^6^, ^25^] The remaining excited state population (non‐reactive pathway) relaxes radiatively (SE, >700 nm, not shown) or non‐radiatively back to the ground state on a much slower timescale. Alongside the rise of J, an additional absorption band occurs around 320–360 nm in KR2, *Er*NaR and *Nm*HR (Figure 2B), <310–340 nm in PR and significantly more blue shifted at <290–320 nm in *Hs*BR. In the latter case, this signature falls into a region of the dominant protein bands which originate from aromatic amino acid residues. Specifically for *Hs*BR it was shown, that the absorption of tryptophans in close proximity to the retinal (and in the right orientation like W86) undergo a dynamic Stark shift due to dipolar coupling with the excited retinal, leading to a complicated dynamics during the excited state decay of the chromophore.[^12^, ^26^] The residual red shoulder of this band—which lasts into the ns timescale—must then be associated with the retinal ground state. In our previous study on KR2,^[11]^ we have assigned the near‐UV signature at 330 nm to a transition from the S_0_ to the S_3_ electronic state of the 13‐*cis* retinal configuration, which is in agreement with the shared lifetime component of the isomerization step (formation of J). Interestingly, in all cases an initial amplitude drop is noticeable in the 1–2 ps timescale, which correlates with the vibrational cooling (transition from J to K) of the isomerized chromophore (Figure S4). This already indicates a strong sensitivity of the SBS transition to the retinal configuration, which is significantly distorted at the initial stages right after the photoisomerization due to the excess energy in the conjugated ethylenic chain. In this context, the relative differences in the amplitude drop between the different rhodopsins could possibly be an indication for different degrees of the initial retinal distortion (and hence cooling, similar to the J−K transition). After this amplitude drop, the signal equilibrates in the ion pumps KR2, *Er*NaR and *Nm*HR and remains constant up to the end of the measurement window (>2 ns), as opposed to the dynamics seen in the proton pumps *Hs*BR and PR (Figure S4), where the signal has substantially decreased. At this stage, the excited state has completely decayed, underlining that the remaining absorption in the near‐UV is indeed not an excited state but a ground state signature.

### Reisomerization Dynamics in the Prototypic Proton‐ and Sodium‐Pumps *Hs*BR, PR and KR2

Since the SBS transition remains present in the ground state dynamics of the photocycle, it is reasonable to follow its evolution on the slower timescales. In this section we aim to establish a more detailed and generalized picture of the SBS signature and its direct correlation with the chromophore configuration, by studying the well characterized microbial rhodopsins *Hs*BR,^[13]^ PR^[14]^ and KR2^[27]^ as model systems by applying combined UV/vis and IR spectroscopy. Furthermore, the focus is set here on the intermediate steps which lead to major changes of the retinal including the protonation steps at the RSB and—most importantly—the reisomerization from 13‐*cis* to all‐*trans*.

**Figure 3:**
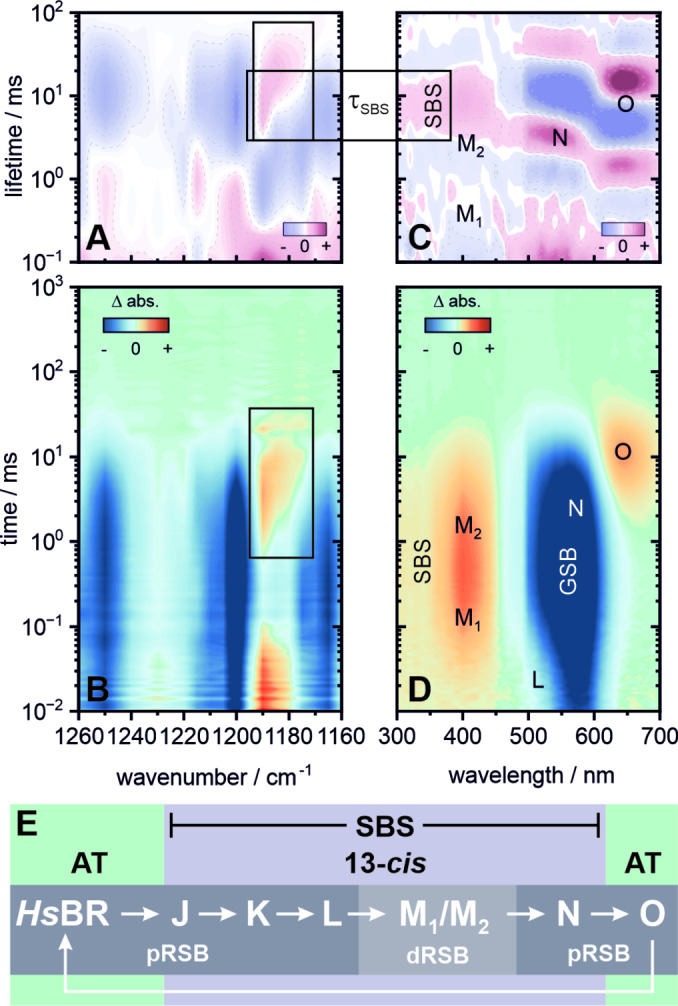
Flash photolysis dataset of *Hs*BR at pH 4. Transient IR data (B) covering the fingerprint region from 1160–1260 cm^−1^ and its corresponding LDM (A). UV/vis data (D) covering 300–700 nm and its corresponding LDM (C). The boxes mark spectral signatures of specific interest and their correlation with the SBS. (E) Schematic overview of the photocycle. An extended lifetime analysis, transient spectra and individual transients are provided in the Supporting Information (Figures S6, S11–S13 and Table S3).

The prototypic (and most well‐studied) photocycle and its nomenclature is derived from *Hs*BR and tied to specific structural and spectral changes of the protein (Figure 3E). With the decay of the vibrationally relaxed ground state intermediate K, the protein subsequently follows the typical sequence of states K−L−M−N−O (Figure 3C, D and E). This includes protonation steps at the RSB (involving the amino acids D85, D96 and D212) as well as the reisomerization of retinal, resulting in the typical spectral shifts seen in the UV/vis (Figure 3D). The latter process specifically occurs with the N−O transition, which also coincides with the decay of the SBS in the near‐UV spectral window (Figure 3 C).

This can be more closely inspected in the complementary mid‐IR region of 1160–1260 cm^−1^, where specifically the vibrational modes around 1240–1255 cm^−1^ (C_12_‐C_13_ stretch, lysine rock), 1200 cm^−1^ (C_14_‐C_15_ stretch) and 1170 cm^−1^ (C_10_‐C_11_ stretch) are common IR markers for the all‐*trans*,15‐*anti* configuration,^[28]^ which is dominantly present in the dark state of light adapted *Hs*BR (and indeed in most microbial rhodopsins—albeit inhomogeneous mixtures are possible in some cases). These bands are found in the transient data of *Hs*BR (Figure 3B) as negative ground state bleach (GSB) signals which persist up to the 10–20 ms range. Here, the only dominant positive signal is found around 1190 cm^−1^, which is commonly attributed to a 13‐*cis*,15‐*anti* marker band.[^29^, ^30^] This band has a more complex kinetic behaviour, being reflected in the additional decay (~0.1 ms) and rise (2–3 ms) components, which are correlated with the deprotonation and reprotonation of the RSB during the formation and decay of the M intermediate. At the last stage of the *Hs*BR photocycle, this band undergoes a slight transient red‐shift from ~1190 cm^−1^ to ~1180 cm^−1^. This frequency has been previously assigned to be characteristic for 13‐*cis*,15‐*syn* retinal (dark adapted *Hs*BR^[29]^ and light adapted *Anabeana* sensory rhodopsin (ASR)^[31]^). Interestingly, the decay of the 1190 cm^−1^ band (and conversely the rise at 1180 cm^−1^) shares a similar lifetime with the final decay of the respective SBS signature (Figures 3A and C), concomitant with the formation of O at 5–6 ms (Figure 3C).

**Figure 4:**
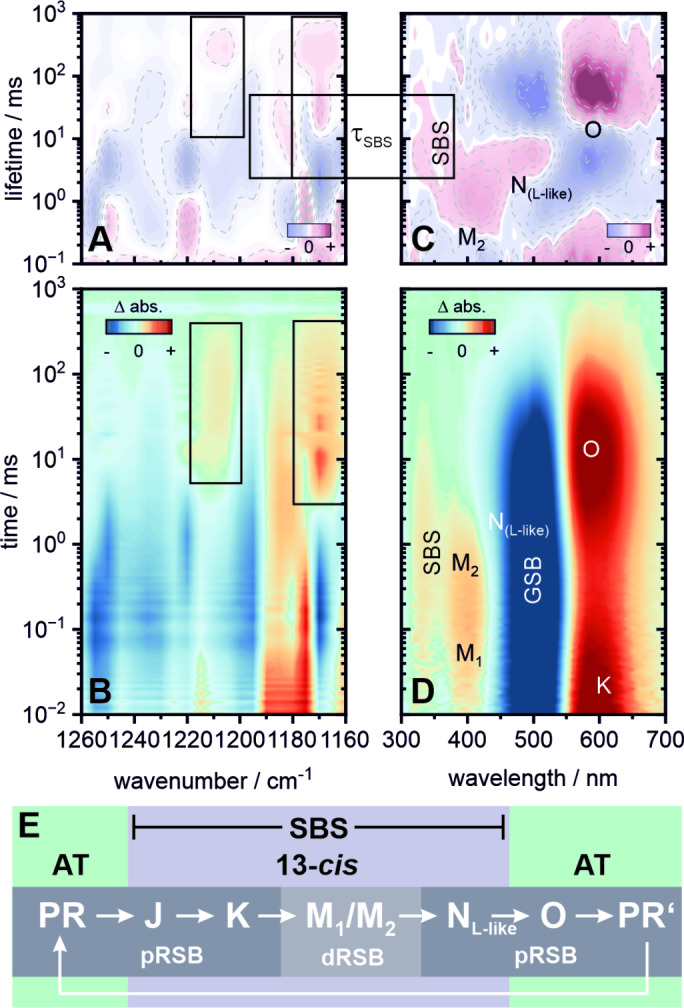
Flash photolysis dataset of PR at pH 8. Transient IR data (B) covering the fingerprint region from 1160–1260 cm^−1^ and its corresponding LDM (A). UV/vis data (D) covering 300–700 nm and its corresponding LDM (C). The boxes mark spectral signatures of specific interest and their correlation with the SBS. The N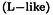 intermediate does not refer to a kinetic equilibrium of the L and N states. It rather reflects the ambiguity of its L‐like spectral position and its N‐like temporal sequence. (E) Schematic overview of the photocycle. An extended lifetime analysis, transient spectra and individual transients are provided in the Supporting Information (Figures S7, S11–S13 and Table S3).

We now take a closer look at another prototypic—but evolutionary distant—proton pump. The photocycle of PR[^18^, ^32^, ^33^] has been studied and characterized soon after its discovery in analogy to *Hs*BR.[^17^, ^34^, ^35^, ^36^, ^37^, ^38^] However, some of the spectroscopic features of PR (at pH >8, its proton‐pumping mode, Figure 4D) are significantly altered compared to its *Hs*BR counterpart (Figure 3D). For the late phase of the photocycle, our dataset (and its evaluation) reveals some inconsistencies with the widely accepted model in current literature (Figure 3E).[^32^, ^39^] In the early spectroscopic studies on PR[^18^, ^32^, ^33^] and mostly ever since, the red‐shifted intermediate was assigned to be N‐like due to the missing IR signature of a reisomerized (all‐*trans*) retinal.[^40^, ^41^] The reisomerization step in the late photocycle was hence linked to a spectrally “silent” kinetic component PR’(O),^[32]^ which contains a twisted all‐*trans* retinal and is followed by its configurational relaxation (and the deprotonation of D97) to restore the initial dark state of PR. The lifetime density analysis (Figure 4C) yet reveals a lifetime component at 1 ms (shared with the late phase of the M‐decay) prior to the formation of the red‐shifted intermediate, which is necessary to describe kinetic changes in the spectral range of 460–515 nm. Interestingly, this spectral range is usually ascribed to L, however judging from the kinetic sequence this contribution is rather N‐like. Due to this ambiguity (and to maintain common nomenclature for the intermediate sequence) we will from now on refer to this component as N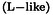 . The decay of the N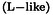 intermediate at 3–4 ms is accompanied with the decay of the SBS signature in the near‐UV, indicating a significant change of the retinal configuration.

This coincides with the decay of the 13‐*cis*,15‐*anti* retinal band at 1175–1190 cm^−1^ and the rise of two additional absorption features at 3 ms—one weak band at ~1210 cm^−1^ and a significantly stronger band at 1170 cm^−1^—in the IR fingerprint region (Figure 4A and B). However, the all‐*trans*,15‐*anti* marker bands at ~1235 cm^−1^ and ~1200 cm^−1^ remain bleached during this transition and are therefore indicative for a not yet fully relaxed retinal. This is in accordance with published work, which reported a distorted retinal in the late photocycle intermediates in PR.[^40^, ^42^]

The origin of the positive difference absorption at 1170 cm^−1^ and 1210 cm^−1^ needs to be discussed. The change of sign of the 1170 cm^−1^ band from negative to positive could indicate an increased oscillator strength of the C_10_‐C_11_ mode as a result of transient changes at and around the chromophore—similar to reports on ASR.^[43]^ An analogous reasoning holds for the 1210 cm^−1^ mode, which lies in the range commonly assigned to the C_8_‐C_9_ stretch.^[28]^ Consequently, this would also imply that the remaining distortions of the retinal chromophore are localized on the middle part of the ethylenic chain. Similar spectral changes have been assigned to an alternative reisomerization pattern which involves the formation of the all‐*trans*,15‐*syn* configuration. This has been widely discussed in the context of *Cr*ChR2 desensitization and more recently as key part of the directed ion transport in sodium pumping rhodopsins.[^44^, ^45^, ^46^, ^47^, ^48^] Taking into account the temporal sequence—e.g. their rise after the decay of the 1190 cm^−1^ band at 3 ms—it is reasonable to assume that these bands are a direct transition product and not the result of a parallelly evolving photocycle originating from inhomogeneities in the dark state of PR. A conclusive assignment, however, requires a closer look into the RSB‐associated IR bands. Nonetheless, the decay of the bleached all‐*trans*,15‐anti bands is multiphasic and occurs on the >100 ms timescale alongside with the decay of the absorption signatures at 1170 cm^−1^ and 1210 cm^−1^ and the red‐shifted intermediate in the UV/vis. This result underlines that the red‐shifted intermediate is indeed O‐like with a distorted all‐*trans* retinal. Consequently, the kinetically necessary decay component previously assigned to the PR’(O) state in several publications,^[32]^ then very likely accounts for the final relaxation of the chromophore distortions as reflected by our IR measurements.

As a light‐driven sodium pump, KR2 is especially interesting due to the transient movement of sodium ions during the photocycle,^[27]^ which ultimately determines the functional mechanism (and hence the kinetics) of several key processes.^[49]^ This is conceptionally easy to grasp if one keeps in mind that both, the ion translocation and the reisomerization of retinal, occur during the presence of O.[^50^, ^51^] Naturally, this (to that date) unique mechanism of cation translocation in microbial rhodopsins has been investigated by many groups utilizing different experimental approaches ranging from steady‐state/time‐resolved crystallography,[^50^, ^51^, ^52^] over optical (UV/vis and vibrational)[^19^, ^21^, ^53^, ^54^, ^55^, ^56^, ^57^, ^58^] and NMR spectroscopy[^59^, ^60^] up to electrophysiology[^61^, ^62^] and computational chemistry.[^11^, ^49^, ^63^] The complexity of events associated with the rise and decay of O and the availability of large amounts of data also lead to many—seemingly contradictory—results, which have been extensively debated ever since.^[20]^ Here we attempt to close this gap and link the SBS dynamics to the vibrational changes of the chromophore, analogously to the analysis of *Hs*BR and PR. As mentioned earlier, we initially reported the SBS as a signature found in KR2, also relying on hybrid QM/MM calculations in order to get a better understanding of the involved energetic landscape for each photointermediate.^[11]^

**Figure 5:**
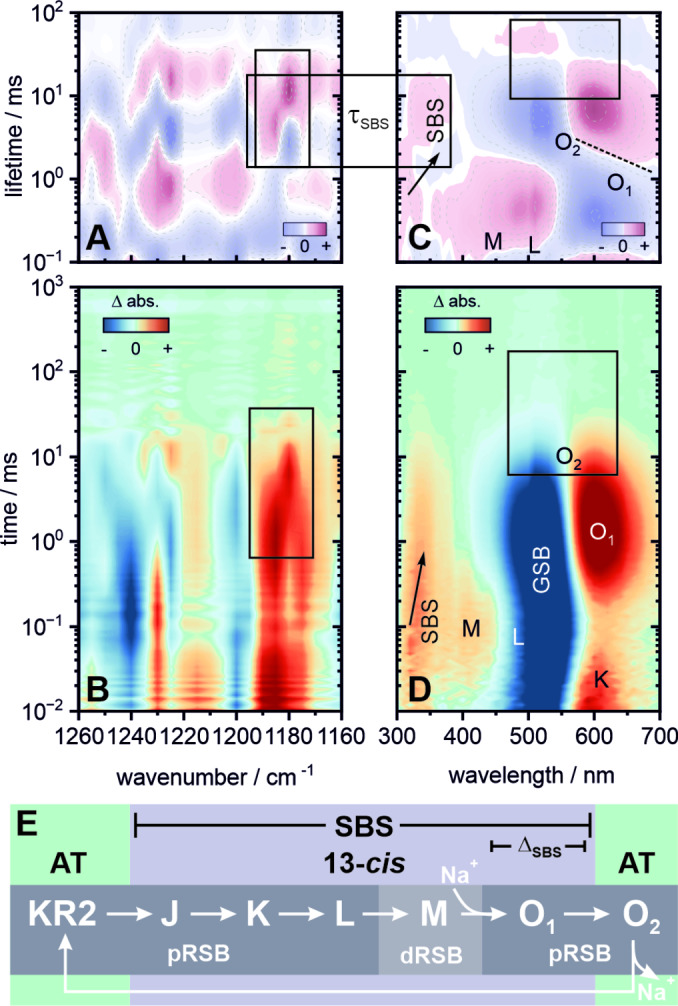
Flash photolysis dataset of KR2 at pH 8. Transient IR data (B) covering the fingerprint region from 1160–1260 cm^−1^ and its corresponding LDM (A). UV/vis data (D) covering 300–700 nm and its corresponding LDM (C). The boxes mark spectral signatures of specific interest and their correlation with the SBS. The tilted dashed line in (C) highlights the spectral inhomogeneity of the O‐decay, suggesting two substates O_1_ and O_2_. The arrows in (C) and (D) indicate the dynamic shift of the SBS by about 20 nm during the formation of O_1_. (E) Schematic overview of the photocycle. An extended lifetime analysis, transient spectra and individual transients are provided in the Supporting Information (Figures S8, S11–S13 and Table S3).

The early photocycle in KR2 is similar to *Hs*BR (Figure 5E). Deprotonation of the RSB by the D116 counterion[^27^, ^50^, ^51^, ^53^, ^54^, ^57^] leads the formation of the M intermediate which is spectrally superimposed with the SBS on the blue side and the L intermediate on the red side (Figure 5D). The latter is in equilibrium with M,^[57]^ as reflected in the spectrally broad distribution (300–510 nm) in the corresponding LDM (Figure 5C) sharing a lifetime of 0.6 ms. The steps associated with ion translocation follow the transition from M to O around 0.6 ms and 2 ms. The decay of O is temporally stretched and spectrally slightly inhomogeneous (tilted line in Figure 5C). This inhomogeneity corresponds to several O‐substates (O_1_ and O_2_) as was suggested in time‐resolved structural data by Skopintsev *et al*.^[50]^ on KR2 as well as kinetic studies by Kato *et al*.^[64]^ and Fujisawa *et al*.^[47]^ on *Ia*NaR from *Indibacter alkaliphilus*. Consequently, the transition from O_1_ to O_2_ at 2 ms marks the molecular switching process and ultimately ensures the vectoriality of sodium transport.[^47^, ^64^] Most notably under the conditions shown here, the transition to O_1_ is also accompanied by a significant spectral redshift (~20 nm) of the SBS which ultimately decays within the transition from O_1_ to O_2_. The dynamic shift of the SBS was not observed in the previously discussed proton pumps *Hs*BR and PR, suggesting at least a slight charge sensitivity of this signature upon sodium uptake. The final decay of O_2_ described by the 6 ms and 20 ms lifetimes then leads to the recovery of the dark state. The latter lifetime accounts for the not yet fully relaxed chromophore and/or environment which is reflected in the residual amplitude of the GSB (see also Figure S12). Similar observations have been reported for other microbial rhodopsins as well, most recently for the inward‐proton pump *Ns*XeR.[^65^, ^66^, ^67^]

**Figure 6:**
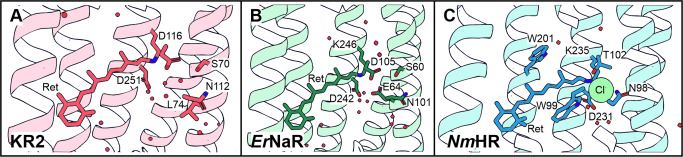
Retinal binding pocket of the Na^+^ pumps KR2 (A) and *Er*NaR (B) and the Cl^−^ pump *Nm*HR (C). The structures are taken from PDB using 6YC3, 8QLF, 7O8F and are rendered with PyMol.

In the IR, the typical bleached bands of all‐*trans*,15‐*anti* retinal^[28]^ at ~1160 cm^−1^, 1200 cm^−1^ and 1240–1255 cm^−1^ as well as the 13‐*cis* retinal product bands at 1185–1190 cm^−1^ are found. The latter is accompanied with a slightly less intense side band around 1175 cm^−1^, which is close to the resonance characteristic for the C_10_‐C_11_ stretch in 13‐*cis* retinal.^[29]^ Such a double band has been reported in a previous study^[68]^ on KR2 at 77 K but was not observed in time‐resolved FTIR experiments at room temperature.^[53]^ Another peculiar set of product bands is centered at 1210–1215 cm^−1^ and 1230 cm^−1^, respectively. This is an uncommon feature, since usually this spectral region is dominated by the bleaching contributions from the C_8_‐C_9_ and the red‐shoulder of the C_12_‐C_13_ stretch modes of all‐*trans* retinal.^[28]^ These differences in the fingerprint region of KR2 (involving C_8_‐C_9_, C_10_‐C_11_ and C_12_‐C_13_ modes) are in line with studies reporting strong twists of the middle part of the chromophore backbone over the majority of the photocycle duration.^[59]^ The 13‐*cis* marker band around 1190 cm^−1^ decays with a lifetime of ~4 ms and undergoes a spectral shift to 1180 cm^−1^ within ~20 ms (Figures 5A and C) while the absorption feature around 1215 cm^−1^ broadens. A similar vibrational signature was observed in *Ia*NaR and proposed to originate from the formation of the all‐*trans*,15‐*syn* configuration.^[48]^ Interestingly, these changes in the IR are preceded by the shift of the SBS from 320 nm to 340 nm at 2 ms and its subsequent decay with a lifetime of ~5–7 ms, which might result from a change of the electrostatic environment around the chromophore right before the reisomerization process. Since a sodium ion is transiently coordinated in the direct proximity of retinal—namely by D116 and N112—during the O intermediates,^[51]^ such an assumption appears reasonable. The nature of this electrostatic interaction remains elusive since the SBS does not show much sensitivity toward changes around the chromophore in the other intermediate states. In our previous study we established that the SBS transition is mainly characterized by the changes of electron densities in the ethylenic chain and therefore omits modulations at and around the RSB.^[11]^ Taking this into account we can speculate that the transient binding of sodium must also lead to a pronounced electrostatic effect on the retinal ethylenic chain. Whether or not the unusual strong twists^[59]^ of the chromophore backbone during the KR2 photocycle play a crucial role here, remains to be further investigated. The final decay of the remaining IR bands follows a similar behavior as seen in the UV/vis, with a stretched decay component of 20 ms and longer—underlining a complex regeneration of the KR2 dark state. The hereby observed sensitivity of the retinal reisomerization dynamics upon transient ion binding reflected in the SBS and the complementary IR bands, motivated us to expand our study to more recently discovered light‐driven ion pumps (Figure 6), which we will discuss in the following section.

### Reisomerization Dynamics in the Novel Light‐Driven Sodium Pump *Er*NaR

Very recently, Podoliak *et al*.^[15]^ reported on the light driven sodium pump *Er*NaR from *Erythrobacter sp*. HL‐111 which has been bioinformatically discovered and structurally characterized. *Er*NaR and KR2 belong to the clade of NDQ rhodopsins, however they are part of different subgroups. Subgroup 1 NDQ rhodopsins (like KR2) possess a leucine or isoleucine at the position analogous to L74 in KR2, yet subgroup 2 NDQ rhodopsins (like *Er*NaR) possess a glutamic acid at this position. The exchange of leucine with a glutamate at this position, leads to a characteristic interaction between E64 (analogue to L74 in KR2) and D105 (analogue to the D116 counterion in KR2) in *Er*NaR which influences the direct RSB environment and ultimately translates into a significantly reduced spectral sensitivity upon changes of pH. This loss of pH‐sensitivity is also reflected structurally in the N101 residue (analogue to N112 in KR2) which does not change its rotameric state as opposed to N112 in KR2 upon fluctuations of the ambient pH‐value.^[52]^ Podoliak *et al*.^[15]^ link these structural nuances to the general protein functionality of NaRs and define a “N‐in” conformation (inactive pump) and “N‐out” conformation (sodium pumping mode),^[15]^ albeit the actual ion translocation pathway in *Er*NaR specifically has not been resolved yet. These structural differences make *Er*NaR an exciting system of study for this work, since we want to test electronic (and vibrational) changes of the chromophore, which are coupled to the transient binding of ions (here Na^+^) in the vicinity of retinal.

**Figure 7:**
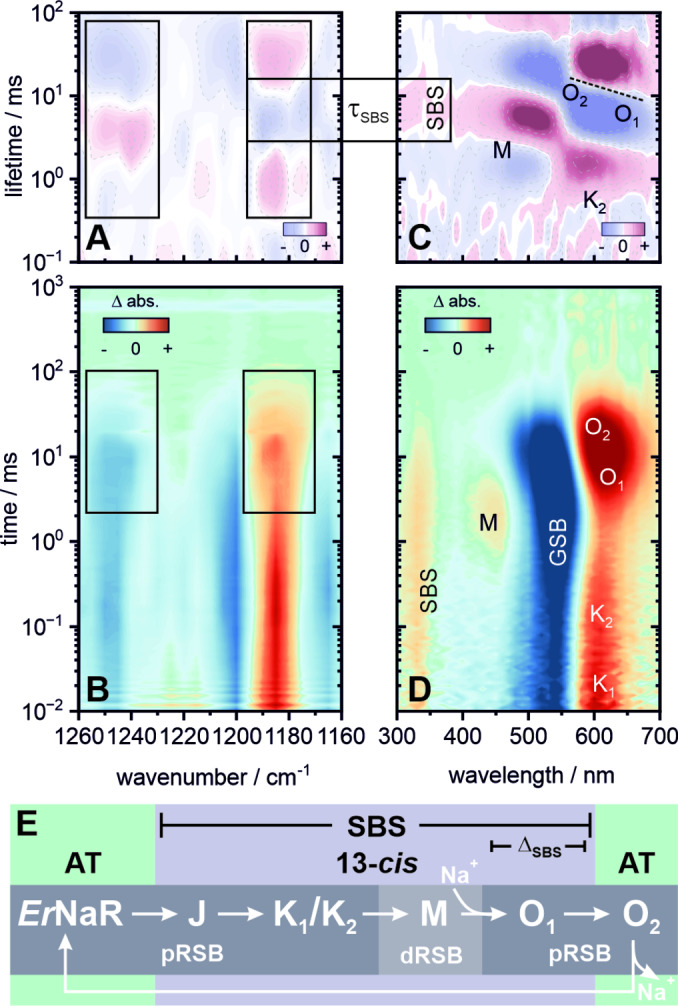
Flash photolysis dataset of *Er*NaR pH 8. Transient IR data (B) covering the fingerprint region from 1160–1260 cm^−1^ and its corresponding LDM (A). UV/vis data (D) covering 300–700 nm and its corresponding LDM (C). The boxes mark spectral signatures of specific interest and their correlation with the SBS. The tilted dashed line in (C) highlights the spectral inhomogeneity of the O‐decay, suggesting two substates O_1_ and O_2_, albeit less pronounced compared to KR2. The SBS undergoes a slight spectral shift, which is less pronounced than in KR2. (E) Schematic overview of the photocycle. An extended lifetime analysis, transient spectra and individual transients are provided in the Supporting Information (Figures S9, S11–S13 and Table S3).

The photocycle of *Er*NaR (Figure 7E) has been discussed in the original publication^[15]^ and shows some nuanced differences compared to KR2. At pH 8 (and 100 mM NaCl), the decay of the thermally equilibrated K intermediate is described multiphasically by two K substates (K_1_ and K_2_) (Figure 7D). This leads to a relatively slow transition to the M intermediate (~2 ms), which is characterized by the transfer of the RSB proton to the D105 counterion. The subsequent reprotonation process of the RSB then leads to a redshift and the rise of O at 6 ms. Closer inspection of the spectral signature suggest a slight inhomogeneity with the red spectral shoulder undergoing faster kinetics than the blue spectral shoulder (Figure 7C). This has been previously assigned to the presence of O_1_ and O_2_ substates in analogy to KR2 and *Ia*NaR.[^47^, ^50^, ^64^, ^69^] Interestingly, the SBS slightly redshifts from 330 nm to 340 nm (Figures 7C and D) at 7–8 ms before it ultimately decays with a 20 ms lifetime simultanous to the rise of O_2_. The shift has to be taken with caution since its strength is probably below or in the order of the experimental spectral resolution (<10 nm), as opposed to the significantly larger change (>20 nm) observed in KR2. Nonetheless, this qualitatively means that during the O intermediate—and hence during the ion translocation—the SBS band is less sensitive in *Er*NaR relative to KR2. The corresponding IR data (Figure 6B) show a similar pattern of bleached bands as found for the other microbial rhodopsins with bands at 1165 cm^−1^, 1200 cm^−1^ and 1250 cm^−1^, stressing that indeed all‐*trans*,15‐*anti* is the prevalent dark state configuration of retinal in *Er*NaR as well. The equivalent product band of the 13‐*cis*,15‐*anti* configuration is located at around 1185 cm^−1^. Of particular interest is the spectral broadening of the 1250 cm^−1^ and 1185 cm^−1^ absorption features at ~10–20 ms which coincide with the decay of the SBS during the O_1_‐O_2_ transition. In analogy to KR2 and *Ia*NaR,[^47^, ^64^, ^69^, ^70^] the presence of these O substates and the spectral changes upon the transition from O_1_ to O_2_ could imply pronounced structural rearrangements around the chromophore and its surrounding which facilitate the directed sodium transport. However, *Er*NaR lacks the characteristic fingerprint bands at ~1170–1180 cm^−1^ and 1210–1220 cm^−1^ found in KR2 and *Ia*NaR, pointing at mechanistic differences in the steps involving sodium translocation.

### Reisomerization Dynamics in the Peculiar Light‐Driven Chloride Pump *Nm*HR

**Figure 8:**
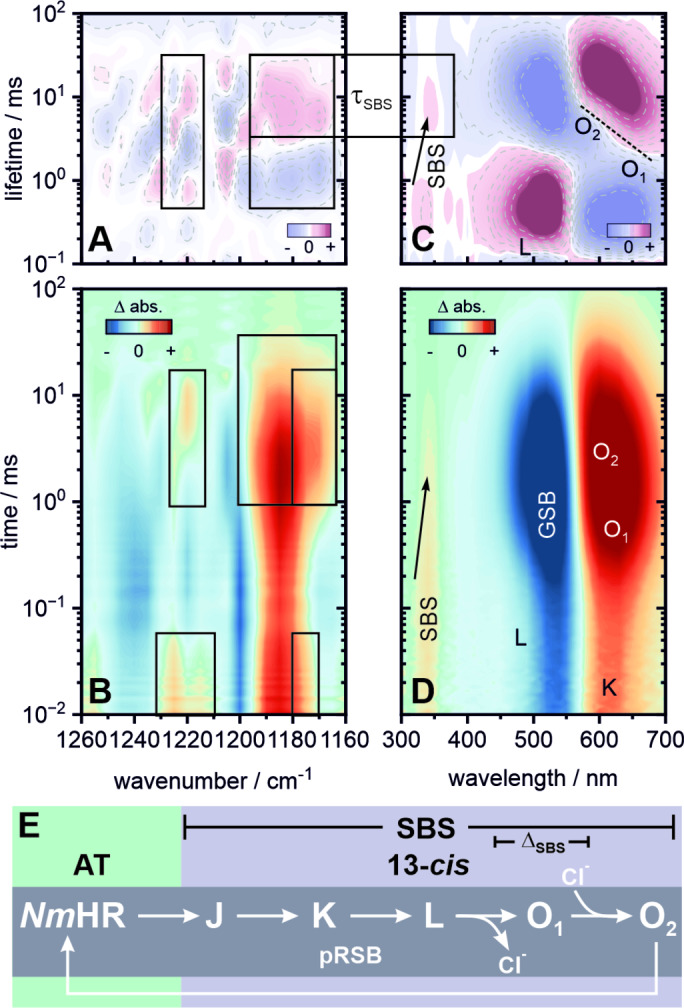
Complete flash photolysis dataset of *Nm*HR pH 7 and 1 M NaCl. Transient IR data (B) covering the fingerprint region from 1160–1260 cm^−1^ and its corresponding LDM (A). UV/vis data (D) covering 300–700 nm and its corresponding LDM (C). The boxes mark spectral signatures of specific interest and their correlation with the SBS. The tilted dashed line in (C) highlights the spectral inhomogeneity of the O‐decay, suggesting two substates O_1_ and O_2_, similar to the sodium pumps KR2 and *Er*NaR. In this case, the arrow highlights the spectral narrowing of the SBS during the formation of O_1_. (E) Schematic overview of the photocycle. An extended lifetime analysis, transient spectra and individual transients are provided in the Supporting Information (Figures S10, S11–S13 and Table S3).

So far, we discussed microbial rhodopsins which transport positive charges either in the form of protons or sodium ions. Here we would like to focus on the inward chloride pump *Nm*HR (or NM−R3 / NmClR)—a more recently discovered member of the HR/ClR class from *Nonlabens marinus*.^[16]^ Despite its functional similarity to halorhodopsins,^[71]^ this peculiar protein has a higher sequence similarity with the light‐driven sodium pump KR2 (35 %) as compared to the anion pumps *Hs*HR (19 %) or *Np*HR (17 %).[^72^, ^73^] More specifically, both KR2 and *Nm*HR share a similar functional motif of NTQ (*Nm*HR) and NDQ (KR2) residues.^[72]^ Instead of the essential D116 counterion in KR2, the bound chloride ion itself takes this role in *Nm*HR. Due to this ambivalent function of the chloride ion—being both counterion and substrate—the photocycle of *Nm*HR (or chloride pumps in more general terms) differs in several key steps from other microbial rhodopsins (Figure 8E).[^72^, ^73^, ^74^, ^75^, ^76^, ^77^]

The isomerization from all‐*trans* to 13‐*cis* retinal in the early stages of the photocycle leads to a loss of hydrogen bonds between the chloride ion and the RSB as well as T102, respectively, and hence an increased mobility of the negative charge.[^73^, ^76^] Instead of following the rotated RSB charge (now directed toward the cytoplasm), the chloride ion diffuses toward T102 during the formation of K.[^73^, ^76^] Once a new hydrogen bond between T102 and Cl^−^ is established on the early μs timescale,^[76]^ the Cl^−^ is pulled toward the RSB which also forces the T102 residue to rotate resulting in the formation of L. This is reflected in the presence of both, the K (~610 nm) and L (~490 nm) states, at the very beginning of the flash photolysis window (Figure  8). The temporal overlap of both intermediates also reveals potential kinetic equilibria at this stage which reflect a still increased mobility of the chloride ion (and a not yet established vectoriality of ion translocation). Therefore, on a timescale of 100–200 μs L decays directly into a red shifted O state (Figures 8C and D). The O state is characterized by a temporal and spectral inhomogeneity^[75]^ which becomes clearly visible in the corresponding LDM (Figure 8C). The changes around the O intermediates (O_1_ and O_2_) also have an influence on the SBS‐signature which shows a multiphasic decay behavior. With the formation of O_1_ at 0.4 ms, the blue shoulder of the SBS starts to vanish (Figure 8D), resulting in a narrower and slightly red‐shifted band centered around 340 nm which ultimately decays with the transition to O_2_ at 5 ms.

The bleached bands in the IR fingerprint region at 1240–1250 cm^−1^, 1200 cm^−1^ and 1160–1170 cm^−1^ are reminiscent of the all‐*trans*,15‐*anti* dark state configuration of retinal. The product band around 1185 cm^−1^ is commonly attributed to the 13‐*cis* configuration of retinal, however some additional features are apparent here. The spectral width of this signature changes in the course of the photocycle with a significant narrowing during the formation of O_1_ (0.4 ms) and another broadening during the transition to O_2_ (5 ms) before it fully decays with a lifetime of approximately 20 ms. In analogy to KR2, a spectral overlap of the C_14_‐C_15_ stretch (being a marker for the 13‐*cis* configuration) and the slightly red‐shifted C_10_‐C_11_ stretch vibrations is apparent. Interestingly, the kinetic pattern observed here also matches the spectral region around 1220 cm^−1^ which is commonly assigned to the C_8_‐C_9_ stretch modes. Those two observations together imply either configurational or electrostatic changes in the middle part of the chromophore, similar to what has been found in KR2. Indeed, Mous *et al*.^[76]^ discussed the electrostatic changes of the retinal due to the direct interactions with a chloride ion. This so‐called anion‐π‐interaction leads to a significant shift of electron density across the molecular plane of retinal with the largest changes close to the RSB and the middle part of the retinal backbone and hence results in a localized polarization effect. The changes around the C_8_‐C_9_ and C_10_‐C_11_ bonds are also kinetically in tune with the changes of the SBS, again underlining a direct relationship between the bonding character along the chromophore chain and this near‐UV transition. On the mechanistic level this implies that the chloride‐RSB‐interaction is removed with the formation of O_1_ and reestablished during the transition to O_2_ when the retinal finally isomerizes back to its initial all‐*trans* configuration. This is in line with serial crystallographic data^[76]^ which connects the release of the initially bound chloride ion toward the cytoplasmic side with the transition from L to O_1_ and the subsequent anion uptake with the formation of O_2_ involving several transient binding sites along the hydrophilic pockets on the extracellular side of the protein.

**Figure 9:**
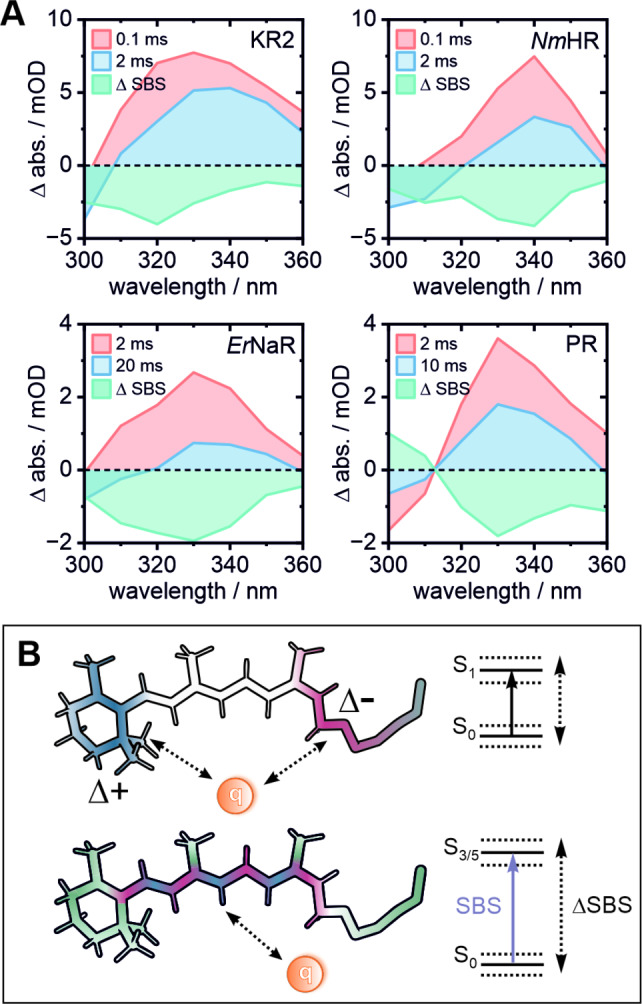
(A) Extracted transient spectra at time points right before (red) and during (blue) the ion translocation step in the cases of KR2, *Er*NaR and *Nm*HR. For PR the time points associated with the N to O transition were chosen. The difference of these spectra (green) reveals transient changes of the SBS band. The ion pumps show a significant decrease of the blue spectral shoulder preceding the decay of the SBS band, which is not observed in the proton pump PR. (B) Schematic comparison of the electrostatic interactions between a generic charge q and the retinal as well as its influence on the S_0_–S_1_ or the S_0_–S_3/5_ transitions. Note that q must not necessarily represent the ion itself. Blue and red represent difference charge densities of the corresponding transitions based on our previous study.^[11]^

We initially hypothesized that the pronounced spectral change of the SBS during the presence of the O_1_ intermediate in KR2 is associated with the transient binding of Na^+^. The direct comparison with the sodium pump *Er*NaR and the chloride pump *Nm*HR should test this hypothesis. Indeed, in all three cases we observe a decrease of amplitude of the blue shoulder of the SBS band during the ion translocating steps (Figure 9A), revealing an underlying bleached absorption band at 300–320 nm. Interestingly, this bleached absorption is also observed in the proton pump PR where it decays alongside the SBS. Yet, in this case the overall spectral shape of the SBS does not change. In our previous work on KR2, the calculated energies for a corresponding SBS of the parent state configuration (all‐*trans*) were estimated to be slightly blue shifted relative to the the transition found for the 13‐*cis* configuration, offering a reasonable explanation for the bleached signals observed here. Overall, these results show a significant difference of the SBS decay dynamics between the ion pumps and the proton pumps (see Figure S13 for *Hs*BR), with the former undergoing a spectral change of the SBS band which precedes the reisomerization step from 13‐*cis* to all‐*trans*. Since the S_0_–S_3/5_ transition corresponds to a redistribution of charge density along the ethylenic chain of retinal (as opposed to the strong CT character of the S_0_–S_1_ transition, Figure 9B), the hereby observed spectral changes are most likely associated with a change of local charge (induced by the ion movement) which mainly affects the retinal backbone, as specifically discussed for *Nm*HR (vide supra).

## Conclusions

With the comparative analysis of selected microbial rhodopsins we attempt to bring together a wide variety of research results from several decades focusing on the (re)isomerization dynamics of retinal in different protein environments covering the fs to s timescale. We emphasize that the use of identical samples for both UV/vis and IR measurements (on the μs‐s timescale) minimizes uncertainties due to sample preparation and thus allows a more direct correlation of electronic and vibrational changes. By applying (quasi) model‐free lifetime distribution analysis (LDA) we were able to investigate complex kinetics and even pinpoint smaller spectral changes or dynamic shifts, that would have otherwise been lost. Despite some protein specific nuances and—in some cases—their inconsistencies with current literature, which are discussed in the respective parts of this work, we would like to highlight the general findings concerning the SBS and its implications on the retinal dynamics:

1. For KR2, the SBS was described as a high energetic (near‐UV) electronic transition from the S_0_ to the S_3_/S_5_ state in 13‐*cis* retinal.^[11]^ Here we show that this marker band is observable in all microbial rhodopsins tested and therefore can serve as a general marker for retinal configuration.
2. The SBS is sensitive to changes in the electrostatic environment of the retinal backbone, as opposed to the S_0_–S_1_ transition which is strongly affected by changes in the CT‐character of the chromophore.
3. This sensitivity also extends to transient ion movements in the direct vicinity of retinal as observed in KR2, *Er*NaR and *Nm*HR.

The extension of the probing range into the near‐UV region (which is often more easily accessible than the mid‐IR range) allows an assessment of characteristic chromophore‐related changes, becoming even more potent if used in tandem with IR spectroscopy (ideally with an identical sample). Especially the spectral sensitivity to steps associated with ion movement across the retinal is a promising and highly desirable probe. Nonetheless, in contrast to the spectroscopic characterization of the SBS, its energetic and transient color‐tuning mechanisms—involving a transiently coordinated charge—are not yet fully understood and offer great opportunities for future computational work.

## Supporting Information

The authors have cited additional references within the Supporting Information.[^77^, ^78^, ^79^, ^80^, ^81^]

## Conflict of Interests

There are no conflicts to declare.

1

## Supporting information

As a service to our authors and readers, this journal provides supporting information supplied by the authors. Such materials are peer reviewed and may be re‐organized for online delivery, but are not copy‐edited or typeset. Technical support issues arising from supporting information (other than missing files) should be addressed to the authors.

Supporting Information

## Data Availability

The data that support the findings of this study are available from the corresponding author upon reasonable request.
