# Supplementary material for: A Detailed View on the (Re)isomerization Dynamics in Microbial Rhodopsins Using Complementary Near‐UV and IR Readouts
Source: Angew Chem Int Ed Engl. 2024 Nov 26;64(4):e202416742. doi: 10.1002/anie.202416742 (PMC11753611; doi:10.1002/anie.202416742)
Supplement: Supplementary file 1 — Supporting Information [file ANIE-64-e202416742-s001.pdf]

Angewandte Chemie
Eine Zeitschrift der Gesellschaft Deutscher Chemiker

Supporting Information

A Detailed View on the (Re)isomerization Dynamics in Microbial Rhodopsins Using Complementary Near-UV and IR Readouts

M. Asido, G. H. U. Lamm, J. Lienert, M. La Greca, J. Kaur, A. Mayer, C. Glaubitz, J. Heberle, R. Schlesinger, K. Kovalev, J. Wachtveitl**

Supporting Information
A Detailed View on the (Re)isomerization Dynamics in Microbial Rhodopsins using Complementary Near-UV and IR Readouts

Marvin Asido,*,+,[a,b] Gerrit H. U. Lamm,+,[a] Jonas Lienert,[a] Mariafrancesca La Greca,[c] Jagdeep Kaur,[d] Anne Mayer,[d] Clemens Glaubitz,[d] Joachim Heberle,[e] Ramona Schlesinger,[c] Kirill Kovalev,[f] Josef Wachtveitl*,[a]

A Detailed View on the (Re)isomerization Dynamics in Microbial Rhodopsins using Complementary Near-UV and IR Readouts

Marvin Asido,*,+,[a,b] Gerrit H. U. Lamm,+,[a] Jonas Lienert,[a] Mariafrancesca La Greca,[c] Jagdeep Kaur,[d] Anne Mayer,[d] Clemens Glaubitz,[d] Joachim Heberle,[e] Ramona Schlesinger,[c] Kirill Kovalev,[f] Josef Wachtveitl*,[a]

Correspondence addressed to: m_asido@mit.edu; wveitl@theochem.uni-frankfurt.de

[a] Dr. M. Asido*, G. H. U. Lamm, J. Lienert, Prof. Dr. J. Wachtveitl*
Institute of Physcial and Theoretical Chemistry, Goethe University Frankfurt, Max-von-Laue Straße 7, 60438 Frankfurt (Main), Germany

[b] Dr. M. Asido*
Present Adress: Department of Chemistry Massachusetts Institute of Technology, 77 Massachusetts Ave, 2-014 Cambridge, Massachusetts 02139, USA

[c] M. La Greca, Dr. R. Schlesinger
Department of Physics, Genetic Biophysics, Freie Universität Berlin, Arnimallee 14, 14195 Berlin, Germany

[d] Dr. J. Kaur, A. Meyer, Prof. Dr. C. Glaubitz
Institute for Biophysical Chemistry and Center for Biomolecular Magnetic Resonance (BMRZ), Goethe University Frankfurt, Max-von-Laue Straße 9, 60438 Frankfurt (Main), Germany

[e] Prof. Dr. J. Heberle
Department of Physics, Experimental Molecular Biophysics, Freie Universität Berlin, Arnimallee 14, 14195 Berlin, Germany

[f] Dr. K. Kovalev
European Molecular Biology Laboratory Hamburg, EMBL Hamburg, c/o DESY, Notkestraße 85, 22607 Hamburg, Germany

[+] These authors contributed equally.

Contents

1. Sample Preparation **S4**
1.1. Sample Conditions . . . S4
1.2. Cuvette Preparation . . . S4

2. Time-resolved UV/vis and IR Spectroscopy **S4**
2.1. Experimental Setups . . . S4
2.1.1. Femtosecond Transient Absorption Spectroscopy . . . S4
2.1.2. Transient Flash Photolysis Spectroscopy . . . S4
2.1.3. Kinetic Analysis of Time-resolved Spectroscopic Data . . . S5
2.2. Supplementary Data and Figures . . . S6
2.2.1. Absorption Spectra . . . S6
2.2.2. Femtosecond Transient UV/vis Spectroscopy Data . . . S7
2.2.3. Summary of the lifetimes obtained from GLA and LDA of the fs-TA datasets . . . S9
2.2.4. Summary of the lifetimes obtained from GLA and LDA of the Flash Photolysis datasets . . . S10
2.2.5. GLA for Transient Flash Photolysis Spectroscopy . . . S11
2.2.6. LDMs for Transient UV/vis Flash Photolysis Spectroscopy . . . S16
2.2.7. LDMs for Transient IR Flash Photolysis Spectroscopy . . . S17
2.2.8. Comparison of Transient UV/vis and IR Flash Photolysis Transients . . . S18
2.2.9. Impact of Sample Preparation on Transient UV/vis Flash Photolysis Spectroscopy Data . . . S19
2.2.10. SBS decay in *Hs*BR . . . S19

3. References **S20**

1. Sample Preparation

1.1. Sample Conditions

Table S1. Summary of the sample conditions used in all experiments.

protein	buffer condition
*Hs*BR	20 mM citrate, 150 mM NaCl (pH 4); (0.05 % DDM in fs-TA)
PR	20 mM HEPES, 150 mM NaCl, 0.05 % DDM (pH 8)
KR2	20 mM HEPES, 150 mM NaCl, 0.05% DDM (pH 8)
*Er*NaR	20 mM Tris-HCl, 100 mM Arg-HCl, 100 mM NaCl, 0.05% DDM (pH 8)
*Nm*ClR	10 mM MOPS, 1 M NaCl, 0.03% DDM (pH 7)

1.2. Cuvette Preparation

All proteins were expressed according to protocols published previously (*Hs*BR,[1] PR,[2] KR2,[3] *Er*NaR,[4] *Nm*ClR[5,6]). Each protein stock solution was adjusted to the respective conditions by washing it three times with the respective buffer solution of specific pH, salt and detergent content (Table S1). For the IR and UV/vis flash photolysis measurements, the samples were then concentrated using a centrifuge (5415 R, Eppendorf, Germany) at 8.6^3 xg. Finally, 12-15 µl of the protein concentrate was placed in the center of a 1 inch CaF_2 window and carefully sealed using a teflon spacer (∼50 µm thickness and ∼1 cm opening) and a second CaF_2 window. By rotating the cuvette and applying even force while tightening the seal, sample leakage and the formation of air bubbles was minimized. For the ultrafast UV/vis measurements, the equilibrated protein solution was adjusted to an OD of ∼0.2 (on a 1 mm optical path length) at a volume of roughly 300 µl with the respective buffer. The sample quality was checked before and after each experiment with an absorption spectrometer (Specord600, Analytik Jena, Germany). See Figure S1 for the corresponding absorption spectra.

2. Time-resolved UV/vis and IR Spectroscopy

2.1. Experimental Setups

2.1.1. Femtosecond Transient Absorption Spectroscopy

Femtosecond transient UV/vis absorption spectroscopy experiments were performed using a home-built pump-probe setup described elsewhere.[4,7] In brief, a fs laser system in which an Amplifier (Spitfire Ace-100F-1K, Spectra-Physics, USA) is seeded by a Ti:Sa oscillator (Mai Tai SP-NSI, Spectra-Physics, USA) and pumped by a Nd:YLF laser (Empower 45, Spectra-Physics, USA) was used. The emitted ultrashort laser pulses (100 fs, 800 nm, 1 kHz repetition rate) were used for generation of the pump pulses in a home-built two-stage noncollinear optical parametric amplifier (NOPA), as well as for white light super continuum generation to obtain the necessary probe pulses. White light generation was achieved via focusing of the laser fundamental (800 nm central wavelength) or the frequency doubled beam (400 nm central wavelength) into a CaF_2-crystal (3 mm). The pump-probe signals were detected using a custom spectrometer consisting of a sensor (MMS UV-VIS II, Carl Zeiss, Germany) and preamplifying electronics (Tec5, Germany). The pump and probe pulses were set to the magic angle (54.7°) configuration to account for anisotropic effects. Furthermore, the sample was continuously moved in a plane perpendicular to the excitation beam to avoid photo-degradation. For all measurements, an excitation energy of 90 nJ/pulse was used.

2.1.2. Transient Flash Photolysis Spectroscopy

Within the study, two different transient flash photolysis spectroscopy setups were used, varying in the accessible probing range. In both, a ns Nd:YAG laser (SpitLight 600, Innolas Laser, Germany) was used to pump an optical parametric oscillator (OPO, preciScan, GWU-Lasertechnik, Germany). The OPO was set to generate pump pulses with a central wavelength of 530-560 nm (depending on the protein) and an average output energy of ∼1.7-2 mJ/cm^2 for the UV/vis and for the IR range, respectively.

In the UV/vis range, absorption changes were probed with the white light provided by a Xenon or a Mercury-Xenon lamp (LC-08, Hamamatsu, Japan) in the range of 300 nm to 700 nm with a stepsize of 10 nm. The white light was guided into two identical monochromators, each equipped with two different gratings (400 nm blaze and 600 nm blaze, both 1200 L/mm). The first monochromator was placed in front of the sample, while the

second one was placed after the sample to reduce effects due to scattering. Scattering effects were further reduced by a rectangular orientation of the pump and the probe beam. A photomultiplier tube (Photosensor H6780-02, Hamamatsu, Japan), mounted directly on the second monochromator, was used to monitor the absorption changes over time. The measured signals were converted into electrical signals and recorded using two oscilloscopes (PicoScope 5244B/D, Pico Technology, England) with overlapping timescales. For each probing wavelength, 30 acquisitions were measured and averaged to increase the S/N ratio. The large raw data files of both oscilloscopes were merged and reduced afterwards. For data reduction forward averaging with a combined linear and logarithmic timescale was applied. The samples were measured in a sandwich cuvette as described previously.[8,9] For our own reference, we also measured the same proteins - but less concentrated - in a 2 x 10 mm Quartz cuvette to exclude significant effects from the sample preparation (see Figure S6 for a comparison in KR2, other samples not shown).

In the IR range, absorption changes were probed using IR light generated by a quantum cascade laser (MIRcat 1100-U2-5086, Daylight Solutions, USA) in the range of 1160 cm^{-1} to 1260 cm^{-1} with a stepsize of 5 cm^{-1}. The signals were detected with an MCT detector (KV104 MCT, Kolmar Tec, USA) connected to the same two oscilloscopes mentioned previously. Additionally, the large raw data files were treated in the same way as for the UV/vis measurements to obtain reduced data files.

2.1.3. Kinetic Analysis of Time-resolved Spectroscopic Data

The time-resolved spectroscopic data was analyzed using OPTIMUS,[10] an analysis software available free-of-charge at www.optimusfit.org. The experimental data was objected to the (quasi) model-free lifetime distribution analysis (LDA), using Tikhonov regularization with 100 regularization factors and 200 evenly distributed lifetime components. The best description is obtained by using the L-curve criterion, which represents the minimum smoothing factor vs residual norm. As a result of the LDA, the lifetime distribution of the individual photointermediate transitions were obtained, which is graphically represented in the corresponding lifetime distribution map (LDM). Together with the 2D representation of the transient data, this allows to pinpoint complex kinetic behavior (e.g. multhiphasic decays) and spectral shifts. The sign of the lifetime distribution amplitude can either correspond to a decay or a rise of a species depending on the transient signal. A positive amplitude in the lifetime distribution corresponds to a decaying species (transient signal is positive) or a population of a new species (transient signal is negative). A negative amplitude in the lifetime distribution corresponds to a decaying species (transient signal is negative) or a population of a new species (transient signal is positive). Note that when using specific lifetimes in the text, we refer to the center of each distribution, however they are explicitly just used for the purpose of comparison with other data (for example specific lifetimes found in the literature). Since global lifetime analysis (GLA) is predominantly used, we also subjected our data to this method to make cross-comparibility with current literature easier. The obtained decay associated spectra (DAS), along with the corresponding lifetimes, yield information about the spectral changes in the course of the photocycle and are similarly interpreted as LDMs.

2.2. Supplementary Data and Figures

2.2.1. Absorption Spectra

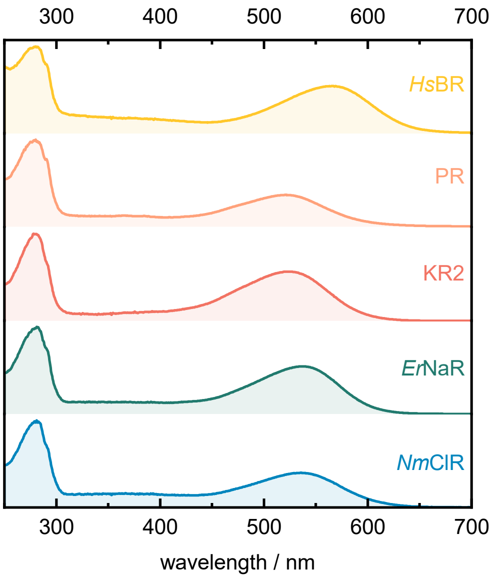

Figure S1. Absorption spectra of all investigated samples in their respective conditions. Intensities have been normalized to the intensity at 280 nm.

2.2.2. Femtosecond Transient UV/vis Spectroscopy Data

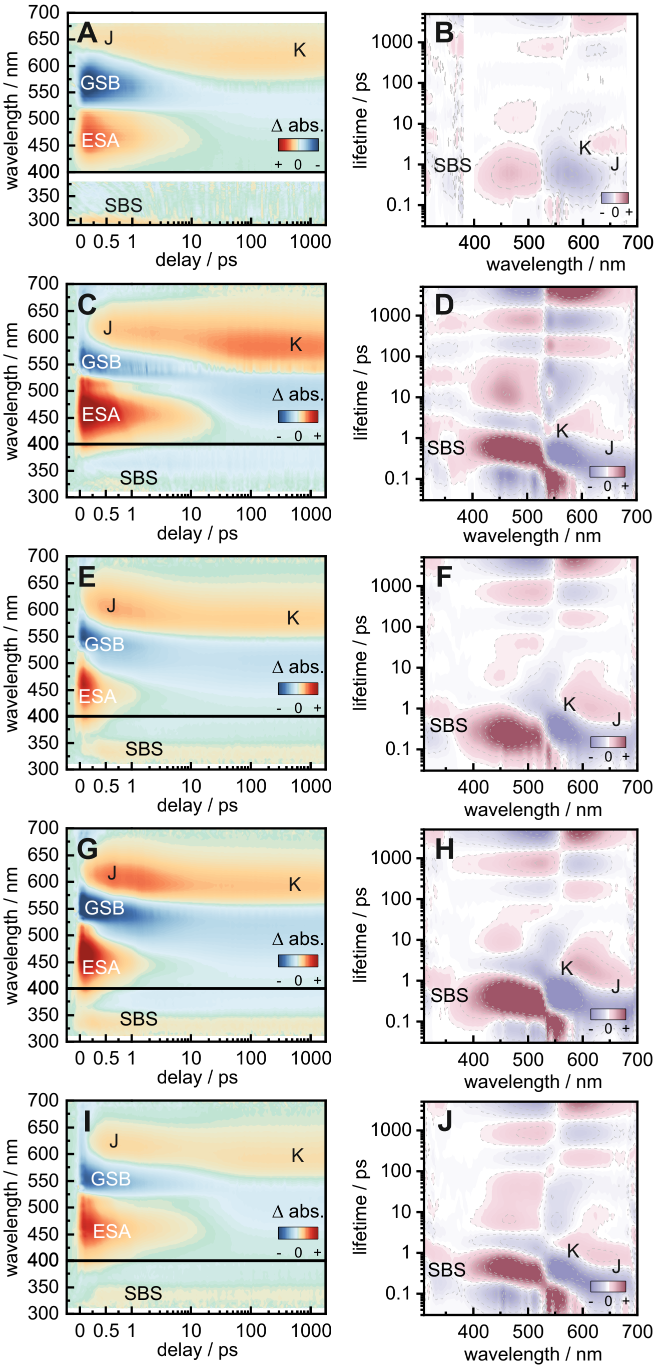

Figure S2. Combined fs-TA datasets and the corresponding lifetime density maps in the spectral range of 290-680 nm for *Hs*BR (A+B) and 310-700 nm for PR (C+D), KR2 (E+F), *Er*NaR (G+H) and *Nm*ClR (I+J). Note that the 310-400 nm region is scaled by a factor of 2 to account for the differences in signal intensity. The data of *Hs*BR is merged from two seperate fs-TA measurements (see experimental section).

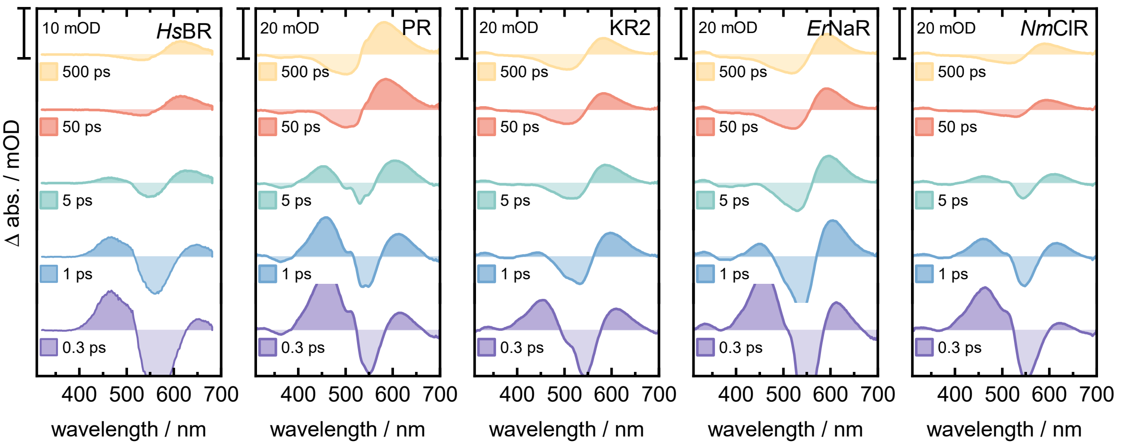

Figure S3. Transient spectra of *Hs*BR in the range of 290-680 nm and PR, KR2, *Er*NaR and *Nm*ClR in the range of 310-700 nm at different time delays.

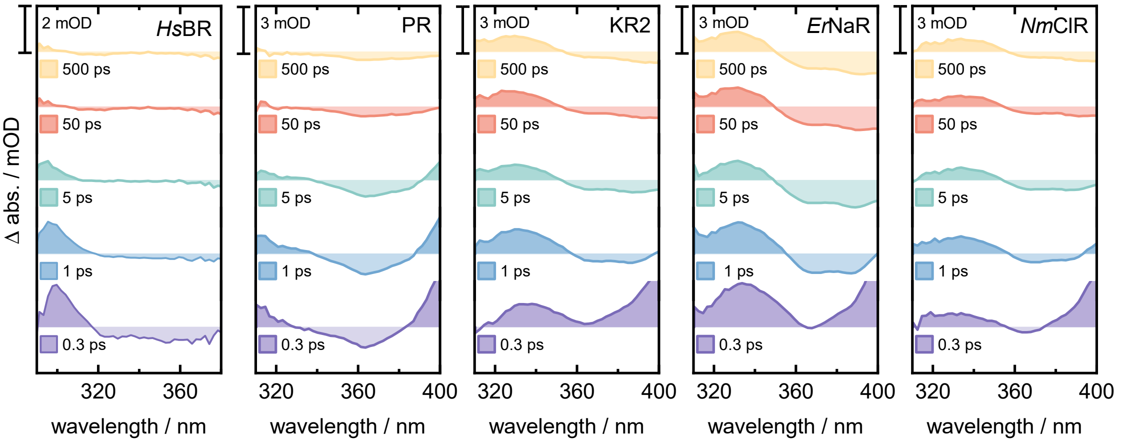

Figure S4. Transient spectra of *Hs*BR in the range of 290-380 nm and PR, KR2, *Er*NaR and *Nm*ClR in the range of 310-400 nm at different time delays. The near-UV absorption feature in *Hs*BR is significantly blue shifted compared to the other samples and therefore superimposed with contributions stemming from the coupling of the chromophore with tryptophan W86 as discussed by Schenkl *et al.*[11,12] Even though the positive absorption band in PR is not as blue shifted as in *Hs*BR, the signal is significantly reduced in the course of the first 500 ps similar to the *Hs*BR case. This is not observed in the ion pumps KR2, *Er*NaR and *Nm*ClR, where the signal remains constant after the initial decay in the first hundred fs, suggesting a difference of the near-UV dynamics between the tested proton pumps and ion pumps on the ultrafast timescale.

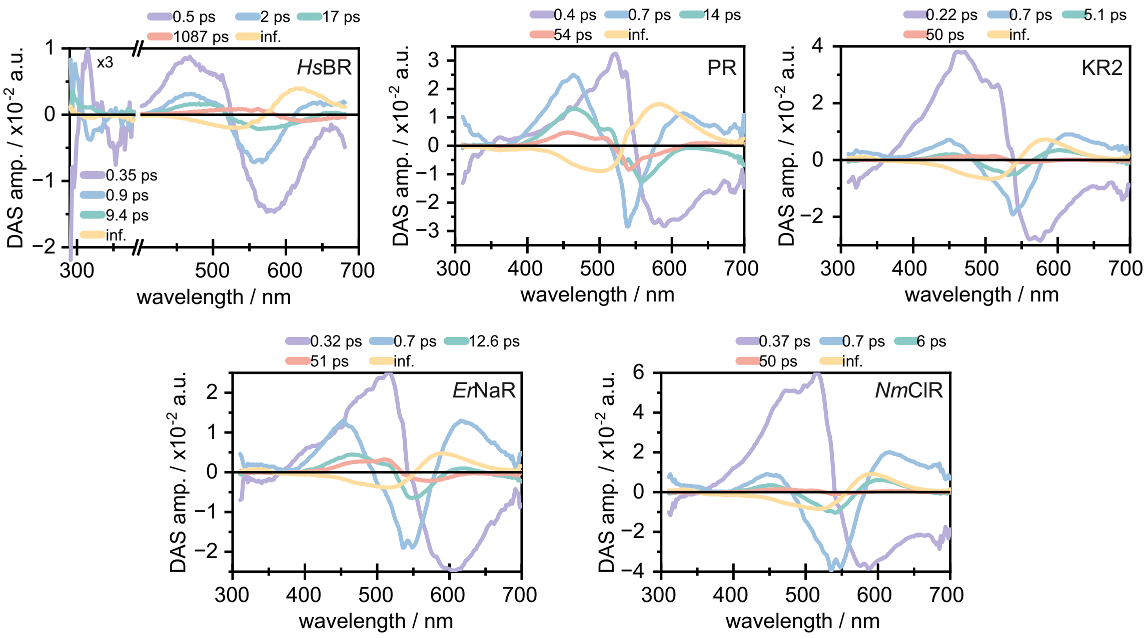

Figure S5. DAS of the fs-TS measurements on all samples. Note that the data of *Hs*BR in the spectral windows from 290-380 (lifetimes on the bottom left) and 400-680 nm (lifetimes on top) have been evaluated separately and were then merged.

2.2.3. Summary of the lifetimes obtained from GLA and LDA of the fs-TA datasets

Table S2. Summary of the global lifetime analysis using five exponential functions to describe the kinetics. Values in brackets are obtained by LDA and correspond to the center of the respective local distribution. A second lifetime in a bracket accounts for lifetime contributions which are not fully resolved in GLA. The last lifetimes (t5) describe the inifinity spectrum due to the residual signal by the end of the measurement window.

		t1 / ps	t2 / ps	t3 / ps	t4 / ps	t5 / ps
*Hs*BR	310-680 nm	0.5 (0.06, 0.4)	2 (1.5, 3)	17 (20)	1087 (1000)	inf.
	290-380 nm	0.35 (0.4)	0.9 (1)	9.4 (7)	/ (1000)	inf.
PR	310-700 nm	0.4 (0.07, 0.3)	0.7 (0.8, 3)	14 (13)	54 (50)	inf
KR2	310-700 nm	0.22 (0.06, 0.22)	0.7 (0.85)	5.1 (2, 7)	50 (40)	inf.
*Er*NaR	310-700 nm	0.32 (0.06, 0.25)	0.7 (0.85)	12.6 (9.5)	51 (45)	inf.
*Nm*ClR	310-700 nm	0.37 (0.07, 0.33)	0.7 (0.9)	6 (2, 7)	50 (40)	inf.

2.2.4. Summary of the lifetimes obtained from GLA and LDA of the Flash Photolysis datasets

Table S3. Summary of the global lifetime analysis using five exponential functions to describe the kinetics. Values in brackets are obtained by LDA and correspond to the center of the respective local distribution. The additional components in t5 are required to account for residual signals (or zero-line) which do not undergo any further changes.

		t1 / ms	t2 / ms	t3 / ms	t4 / ms	t5 / ms
*Hs*BR	UV/vis	0.08 (0.09)	1.1 (1.1)	2.3 (2)	5.4 (8)	11.4 (17)
	IR	0.08 (0.08)	0.9 (0.8)	2.6 (2.5)	5.3 (6)	12.5 (15)
PR	UV/vis	0.07 (0.08)	1 (1.4)	3.4 (4)	19.7 (20)	96 (100)
	IR	0.05 (0.08)	0.7 (0.6)	3 (3)	19 (25)	90 (100, 250)
KR2	UV/vis	0.03 (0.04)	0.4 (0.6)	1.6 (2)	5 (7)	23 (20)
	IR	0.05 (0.08)	0.3 (0.3)	1.5 (1)	2.5 (3)	13 (20)
*Er*NaR	UV/vis	0.07 (/)	0.5 (/)	2.2 (2)	5.2 (6)	22 (25)
	IR	0.07 (/)	0.1 (0.1)	1.5 (1.5)	4.8 (6)	25 (30, 250)
*Nm*ClR	UV/vis	0.08 (0.07)	0.4 (0.4)	1.9 (2)	5.5 (8)	23 (25)
	IR	0.06 (/)	0.74 (0.6)	2.2 (2)	4.5 (5)	19 (15, 100, 250)

2.2.5. GLA for Transient Flash Photolysis Spectroscopy

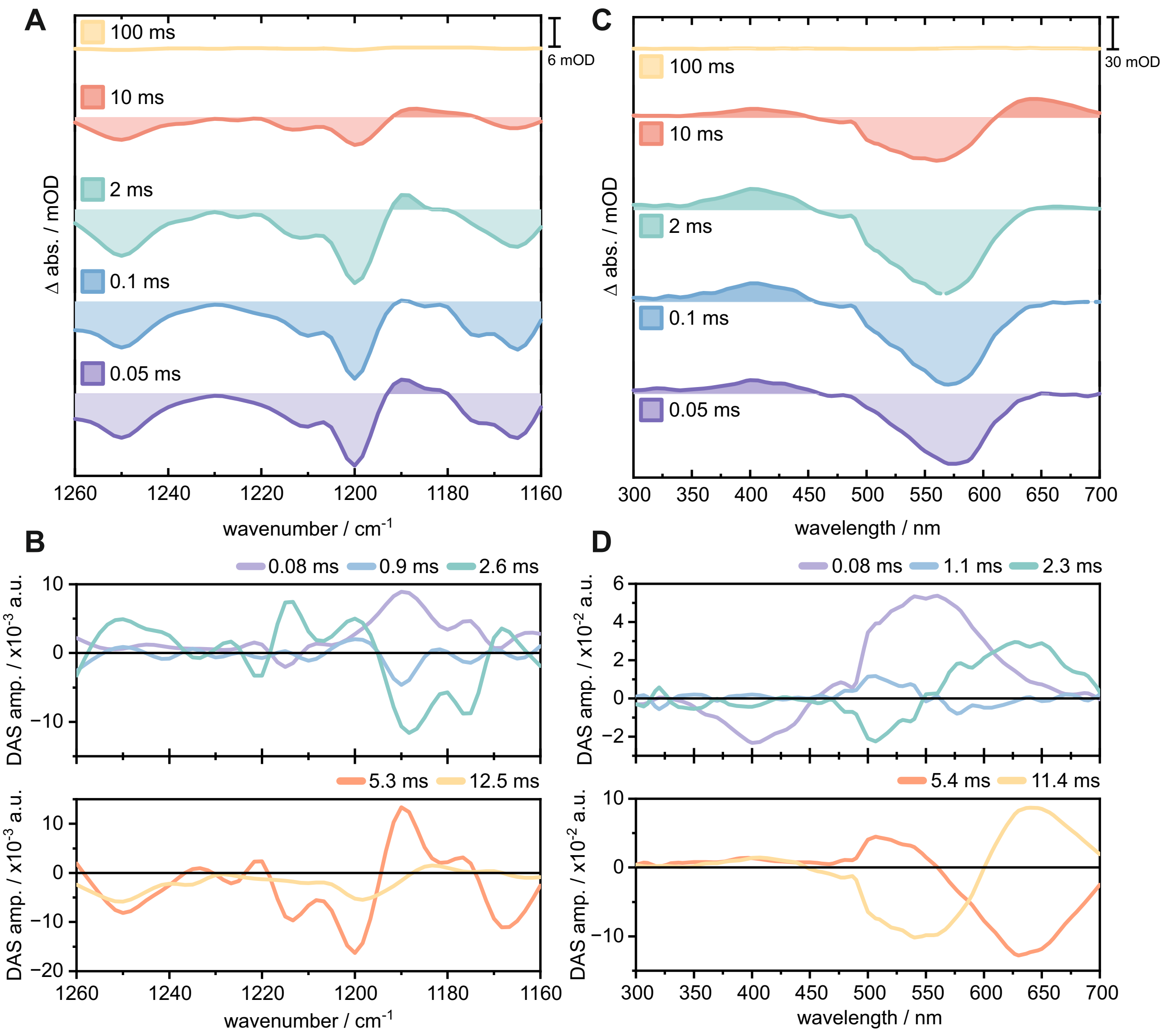

Figure S6. Flash photolysis measurement of *Hs*BR. Transient spectra of the IR measurement (A) and the corresponding DAS (B) in the mid-IR fingerprint region. Transient spectra of the UV/vis measurement (C) and the corresponding DAS (D) covering 300-700 nm..

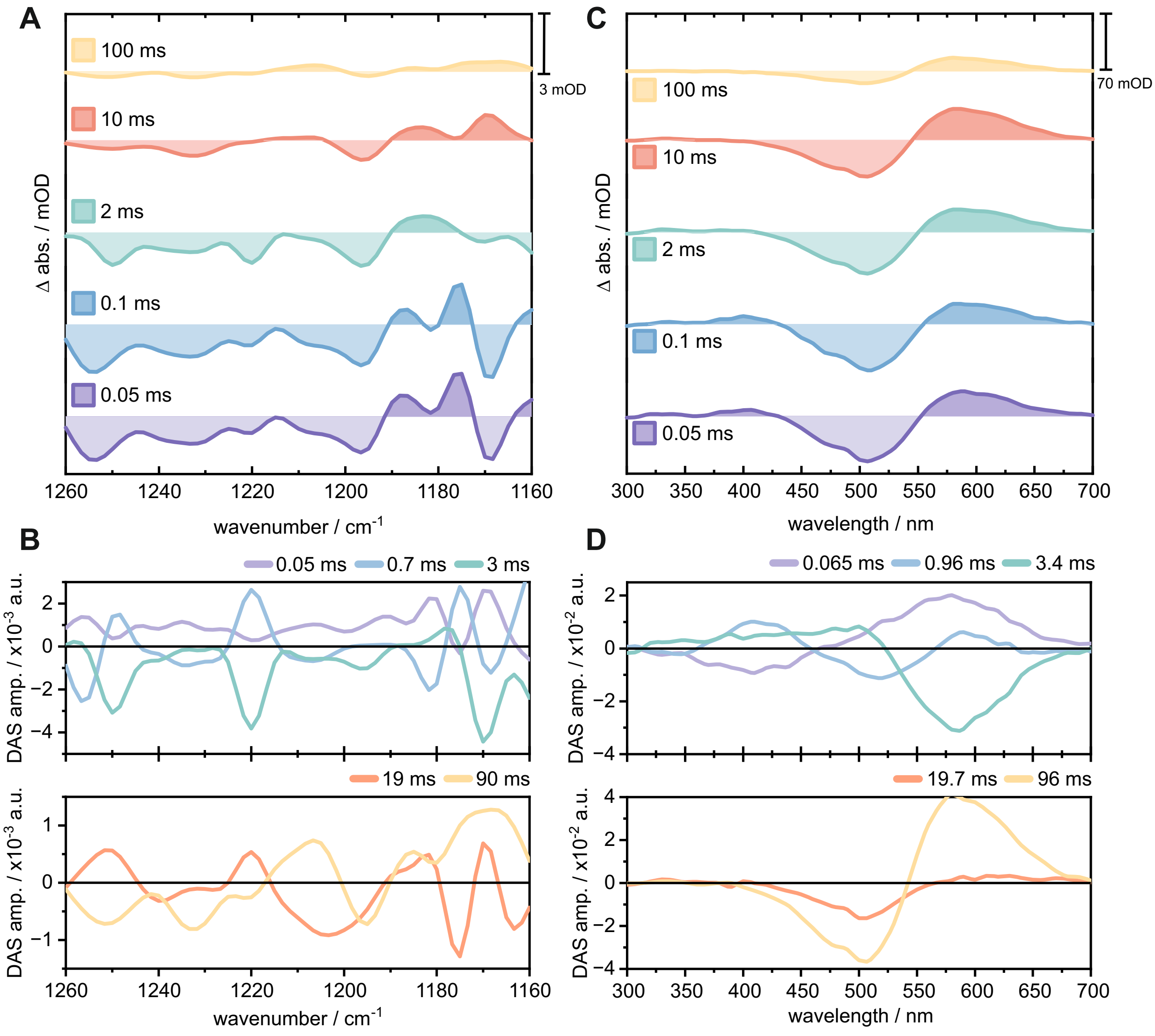

Figure S7. Flash photolysis measurement of PR. Transient spectra of the IR measurement (A) and the corresponding DAS (B) in the mid-IR fingerprint region. Transient spectra of the UV/vis measurement (C) and the corresponding DAS (D) covering 300-700 nm.

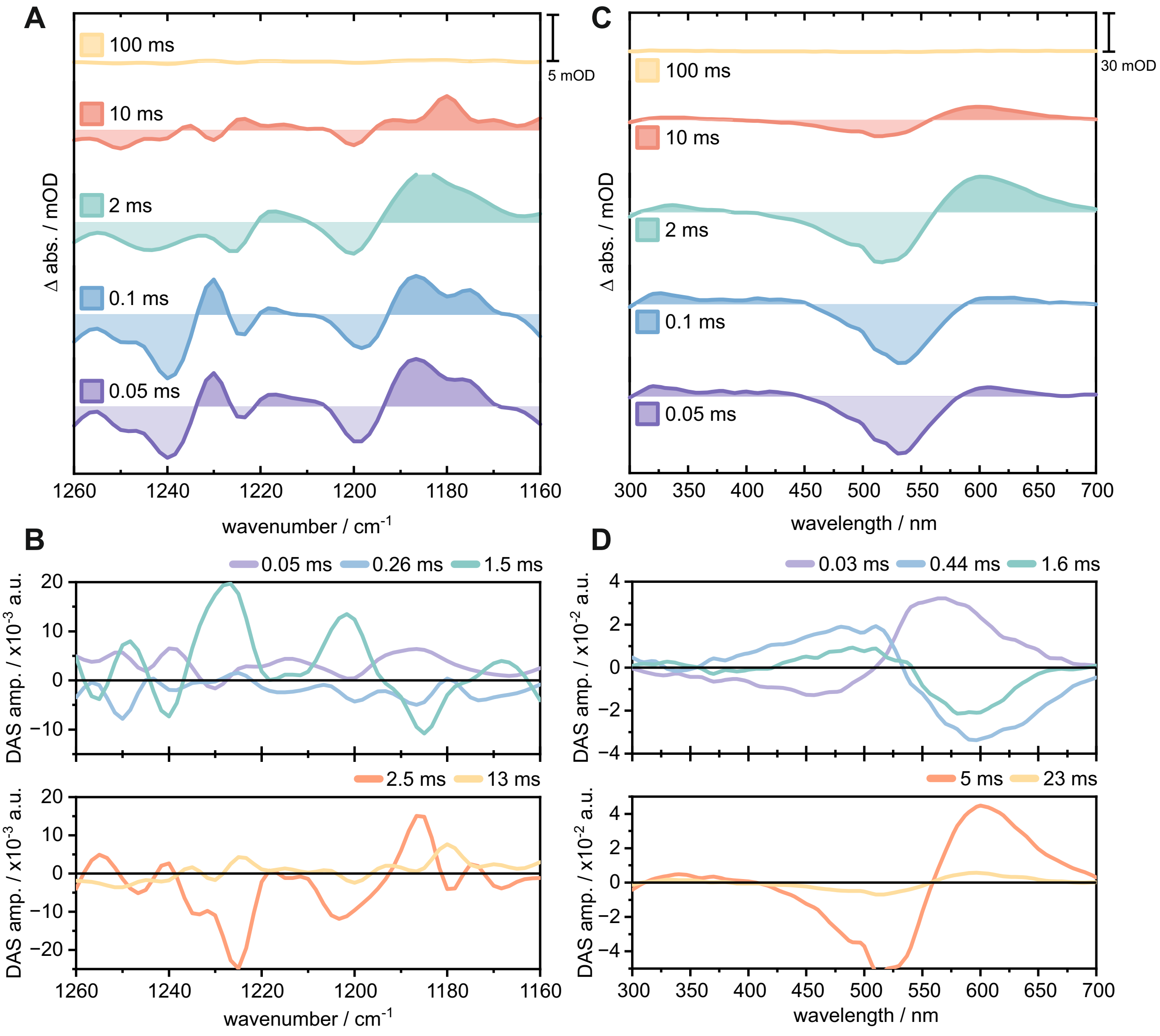

Figure S8. Flash photolysis measurement of KR2. Transient spectra of the IR measurement (A) and the corresponding DAS (B) in the mid-IR fingerprint region. Transient spectra of the UV/vis measurement (C) and the corresponding DAS (D) covering 300-700 nm.

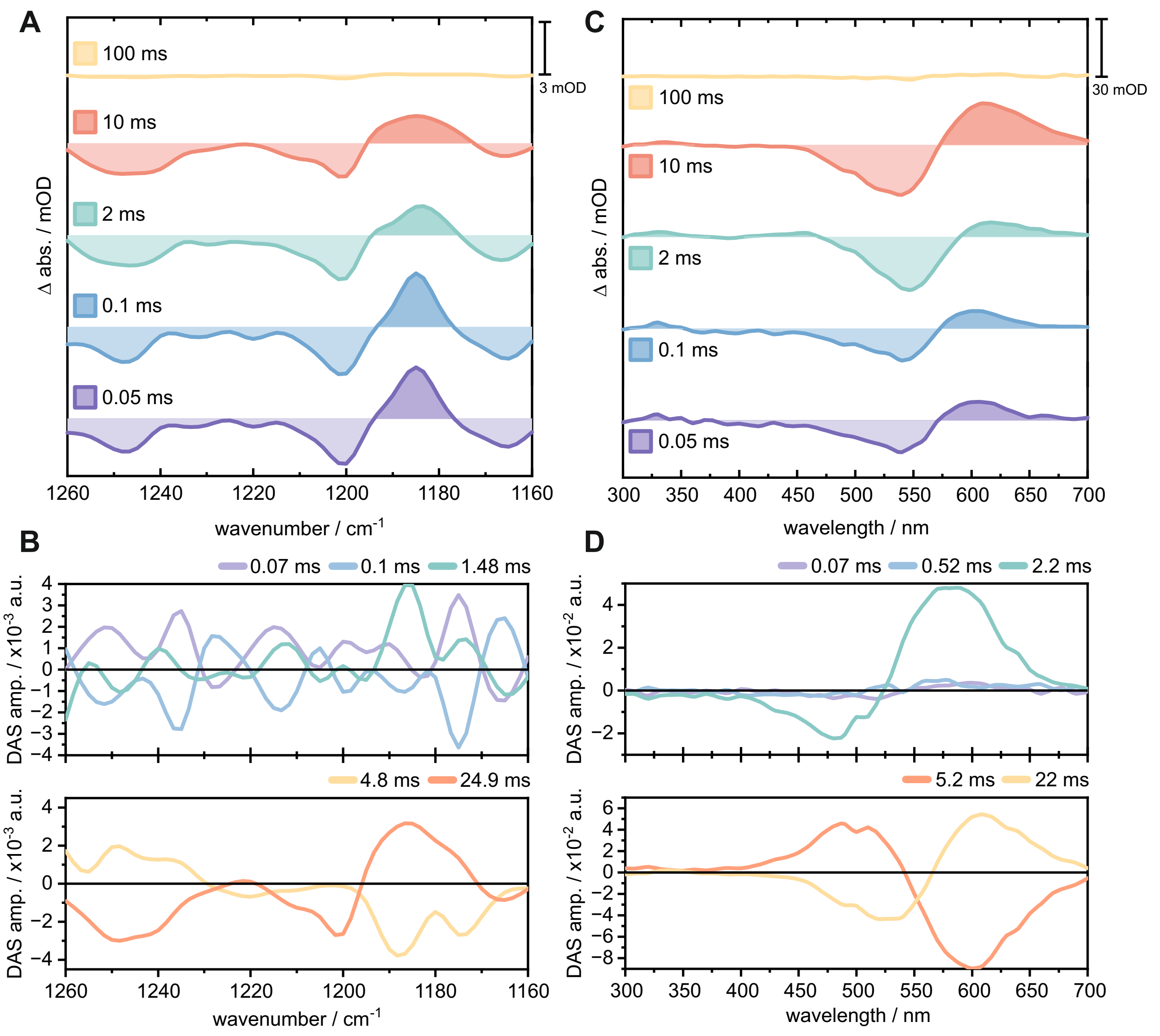

Figure S9. Flash photolysis measurement of *Er*NaR. Transient spectra of the IR measurement (A) and the corresponding DAS (B) in the mid-IR fingerprint region. Transient spectra of the UV/vis measurement (C) and the corresponding DAS (D) covering 300-700 nm.

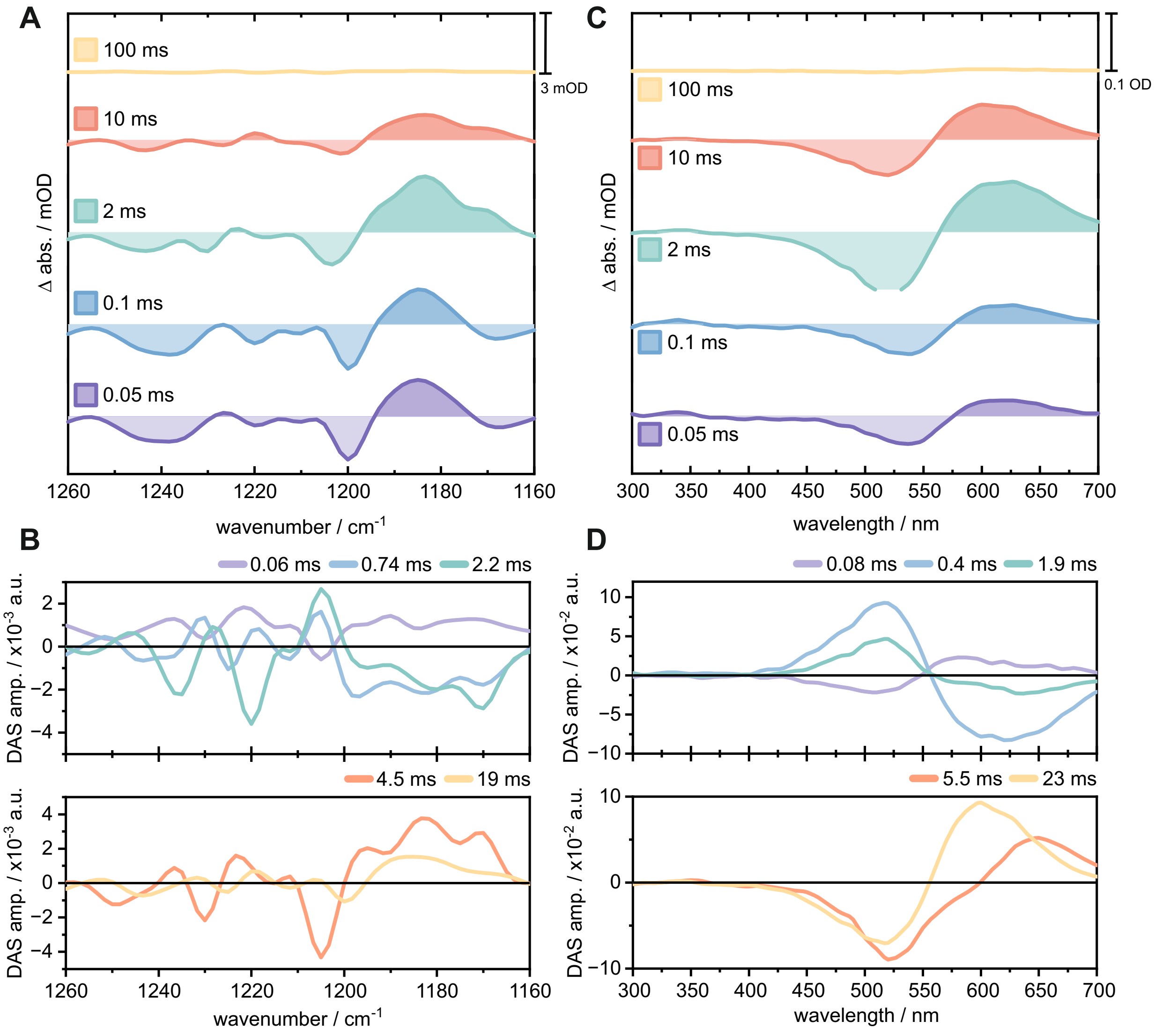

Figure S10. Flash photolysis measurement of *Nm*CIR. Transient spectra of the IR measurement (A) and the corresponding DAS (B) in the mid-IR fingerprint region. Transient spectra of the UV/vis measurement (C) and the corresponding DAS (D) covering 300-700 nm.

2.2.6. LDMs for Transient UV/vis Flash Photolysis Spectroscopy

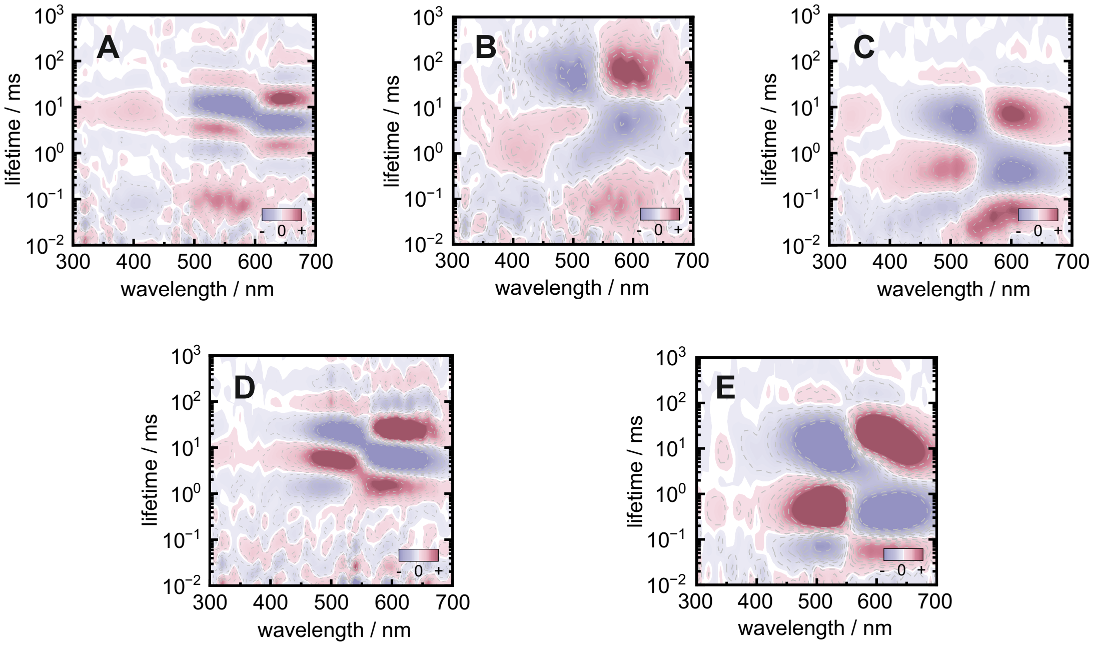

Figure S11. Extended LDMs of the UV/vis flash photolysis datasets. (A) *Hs*BR, (B) PR, (C) KR2, (D) *Er*NaR, (E) *Nm*ClR.

2.2.7. LDMs for Transient IR Flash Photolysis Spectroscopy

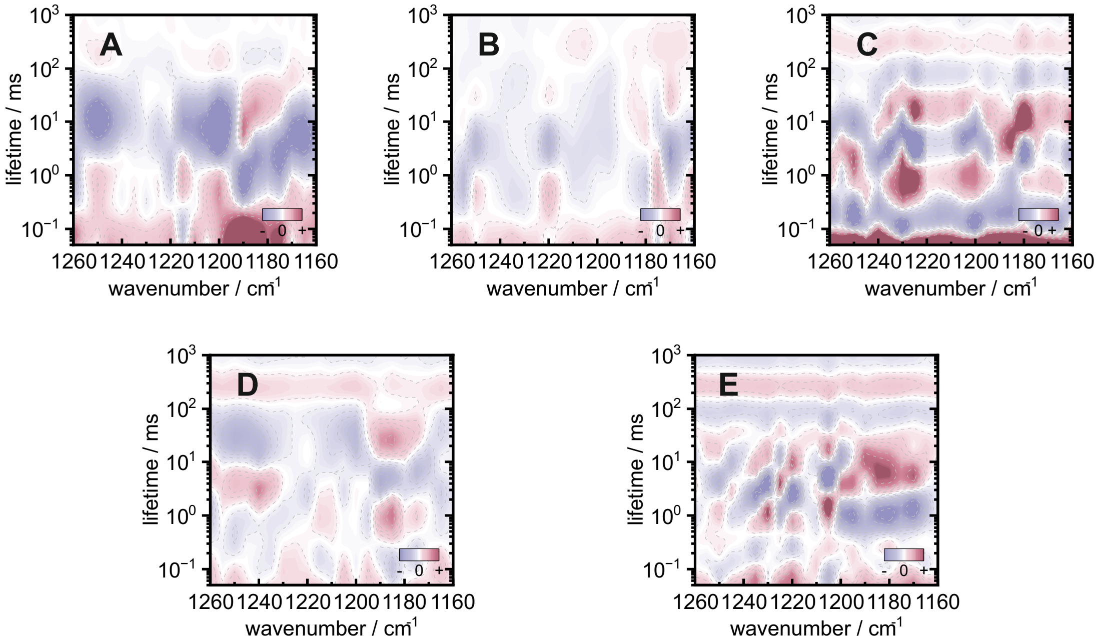

Figure S12. Extended LDMs of the IR flash photolysis datasets. (A) *Hs*BR, (B) PR, (C) KR2, (D) *Er*NaR, (E) *Nm*ClR.

2.2.8. Comparison of Transient UV/vis and IR Flash Photolysis Transients

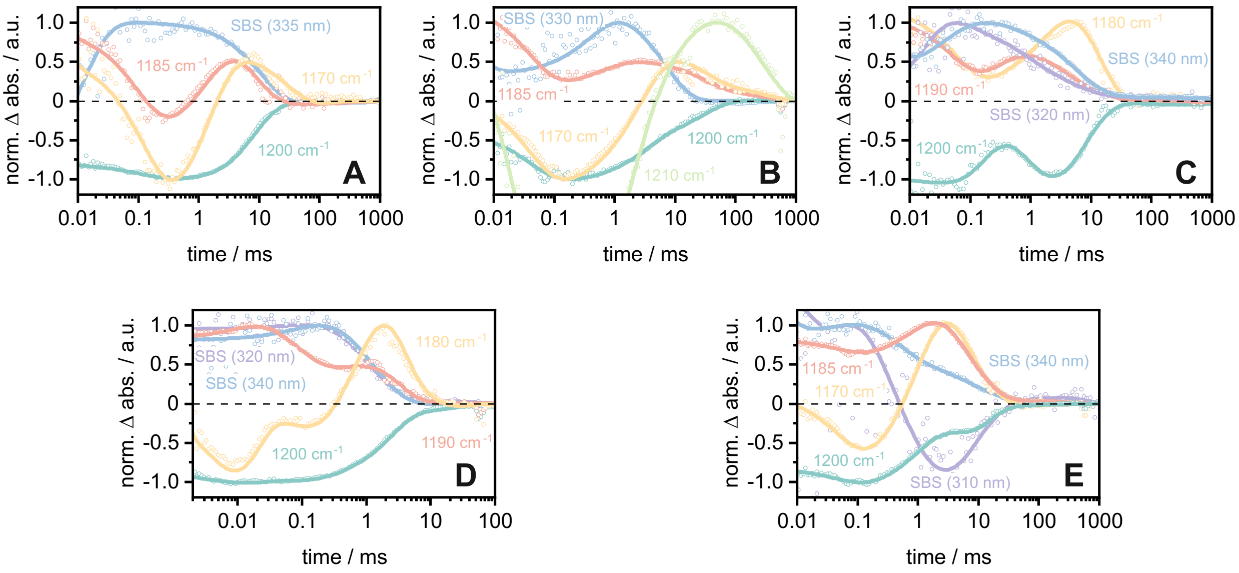

Figure S13. Selected transients extracted from the UV/vis and IR flash photolysis datasets. (A) *Hs*BR, (B) PR, (C) KR2, (D) *Er*NaR, (E) *Nm*ClR.

2.2.9. Impact of Sample Preparation on Transient UV/vis Flash Photolysis Spectroscopy Data

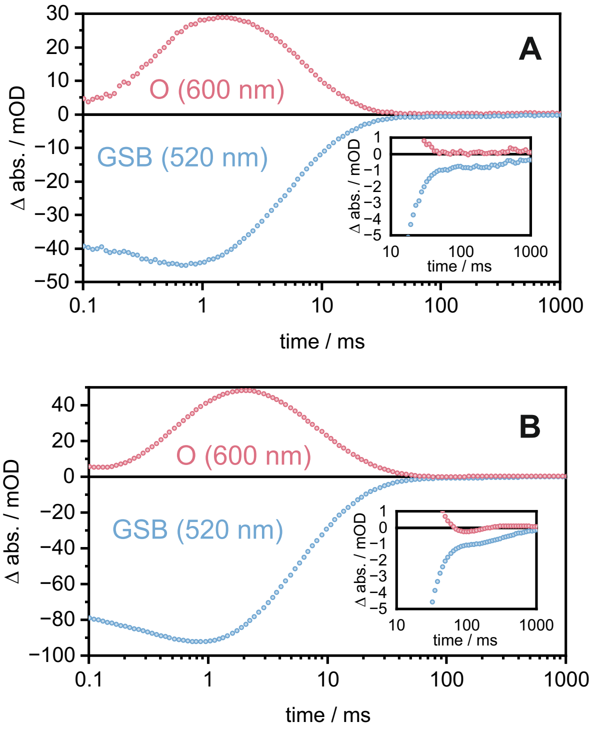

Figure S14. Comparison of selected transients in the UV/vis region. (A) IR sample conditions and (B) less concentrated sample in 2x10 mm quartz cuvette. The inlets are a zoom into the region of interest, which shows a residual bleach signal at >100 ms.

2.2.10. SBS decay in *Hs*BR

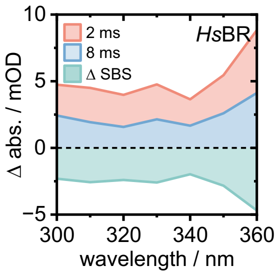

Figure S15. Selected transient spectra of *Hs*BR UV/vis measurement at 2 ms and 8 ms. The difference shows that the SBS is just decaying without any change of the spectral shape.

3. References

References

[1] A. J. Mason, S. L. Grage, S. K. Straus, C. Glaubitz, A. Watts, *Biophysical Journal* **2004**, *86*, 1610.

[2] K. Neumann, M.-K. Verhoefen, I. Weber, C. Glaubitz, J. Wachtveitl, *Biophysical Journal* **2008**, *94*, 4796.

[3] K. Kovalev, V. Polovinkin, I. Gushchin, A. Alekseev, V. Shevchenko, V. Borshchevskiy, R. Astashkin, T. Balandin, D. Bratanov, S. Vaganova, A. Popov, V. Chupin, G. Büldt, E. Bamberg, V. Gordeliy, *Science Advances* **2019**, *5*, eaav2671.

[4] E. Podoliak, G. H. U. Lamm, E. Marin, A. V. Schellbach, D. A. Fedotov, A. Stetsenko, M. Asido, N. Maliar, G. Bourenkov, T. Balandin, C. Baeken, R. Astashkin, T. R. Schneider, A. Bateman, J. Wachtveitl, I. Schapiro, V. Busskamp, A. Guskov, V. Gordeliy, A. Alekseev, K. Kovalev, *Nature Communications* **2024**, *15*, 3119.

[5] K. Kim, S.-K. Kwon, S.-H. Jun, J. S. Cha, H. Kim, W. Lee, J. F. Kim, H.-S. Cho, *Nature Communications* **2016**, *7*, 12677.

[6] S. Oldemeyer, M. La Greca, P. Langner, K.-L. Lê Công, R. Schlesinger, J. Heberle, *Journal of the American Chemical Society* **2024**, *146*, 19118.

[7] T. Saßmannshausen, A. Kunz, N. Oberhof, F. Schneider, C. Slavov, A. Dreuw, J. Wachtveitl, H. A. Wegner, *Angewandte Chemie International Edition* **2024**, *63*, e202314112.

[8] M. Asido, P. Eberhardt, C. N. Kriebel, M. Braun, C. Glaubitz, J. Wachtveitl, *Physical Chemistry Chemical Physics* **2019**, *21*, 4461.

[9] P. Skopintsev, D. Ehrenberg, T. Weinert, D. James, R. K. Kar, P. J. M. Johnson, D. Ozerov, A. Furrer, I. Martiel, F. Dworkowski, K. Nass, G. Knopp, C. Cirelli, C. Arrell, D. Gashi, S. Mous, M. Wranik, T. Gruhl, D. Kekilli, S. Brünle, X. Deupi, G. F. X. Schertler, R. M. Benoit, V. Panneels, P. Nogly, I. Schapiro, C. Milne, J. Heberle, J. Standfuss, *Nature* **2020**, *583*, 314.

[10] C. Slavov, H. Hartmann, J. Wachtveitl, *Analytical Chemistry* **2015**, *87*, 2328.

[11] S. Schenkl, F. van Mourik, G. van der Zwan, S. Haacke, M. Chergui, *Science* **2005**, *309*, 917.

[12] S. Schenkl, F. van Mourik, N. Friedman, M. Sheves, R. Schlesinger, S. Haacke, M. Chergui, *Proceedings of the National Academy of Sciences* **2006**, *103*, 4101.
